# Supplementary figures and images for: SNARE mimicry by the CD225 domain of IFITM3 enables regulation of homotypic late endosome fusion
Source: EMBO J. 2024 Dec 9;44(2):534–62. doi: 10.1038/s44318-024-00334-8 (PMC11730294; doi:10.1038/s44318-024-00334-8)

Figure 1B

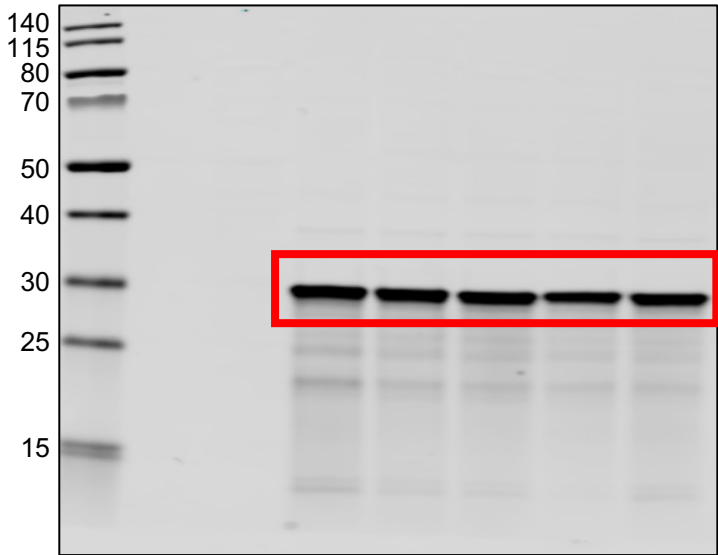

HA

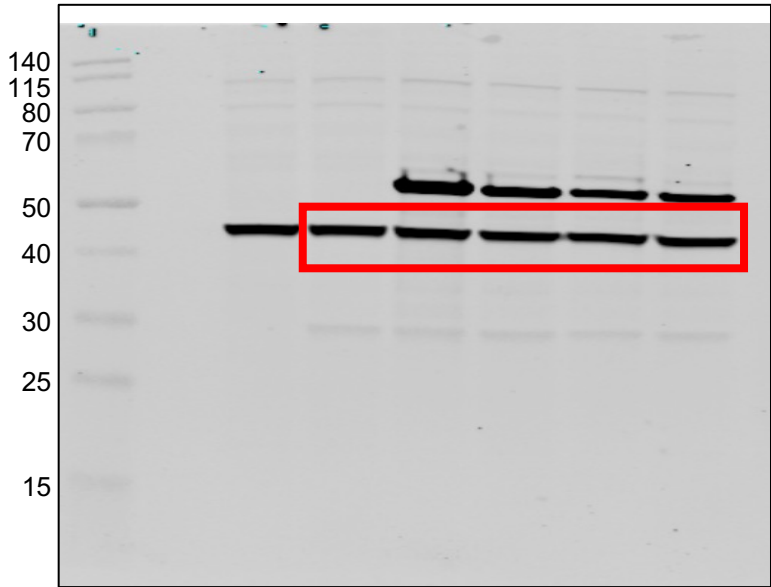

Actin

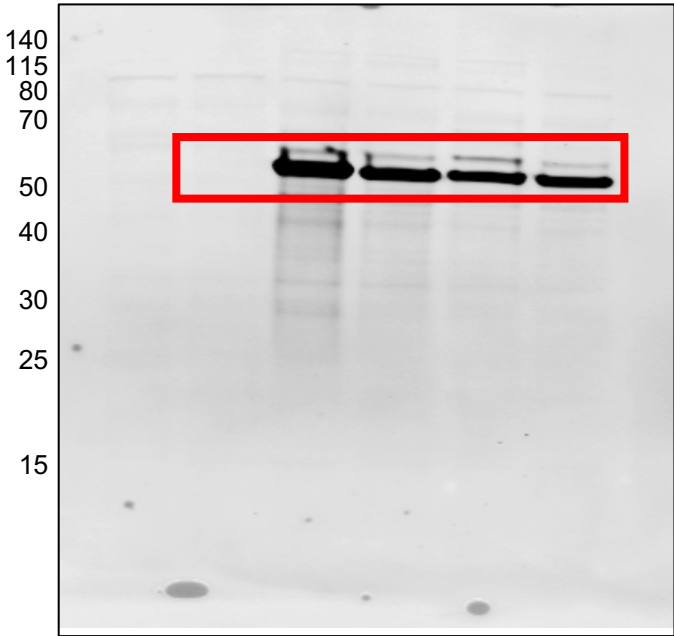

FLAG

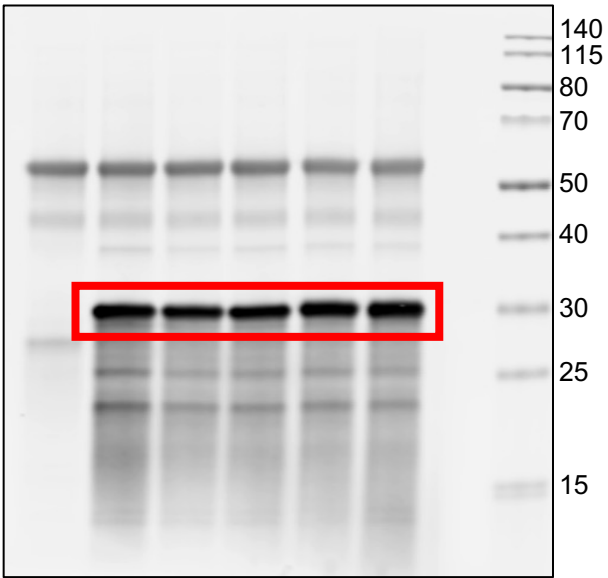

HA

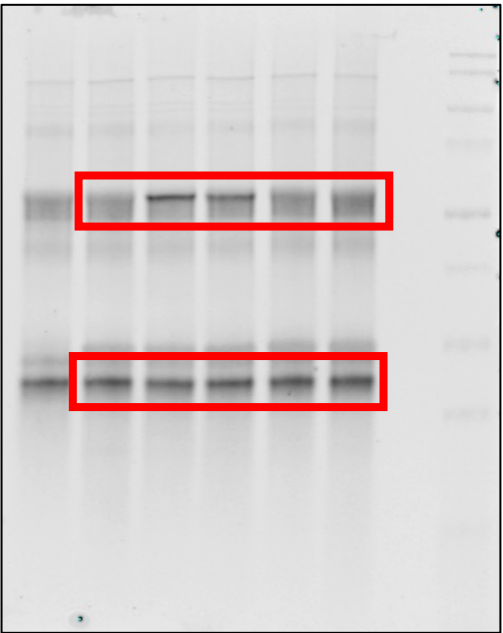

FLAG

Ig Light Chain

Supplement: Supplementary file 3 — Source data Fig. 1 [file 44318_2024_334_MOESM3_ESM.zip › Figure 1/Figure 1B Blots.pdf]

Figure 2E

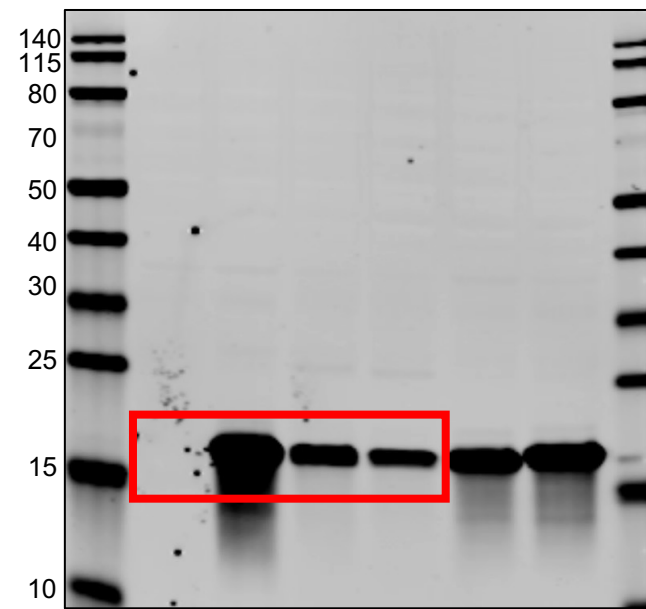

FLAG

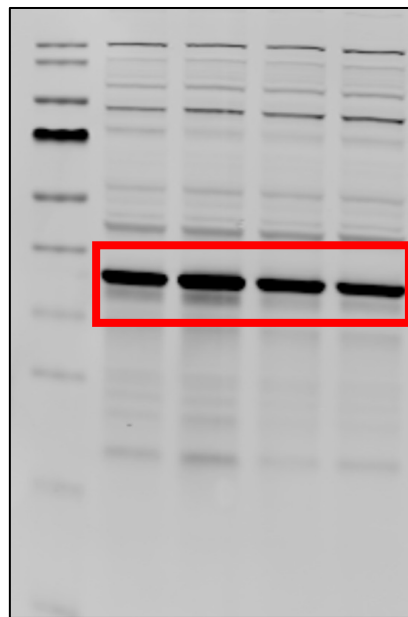

HA

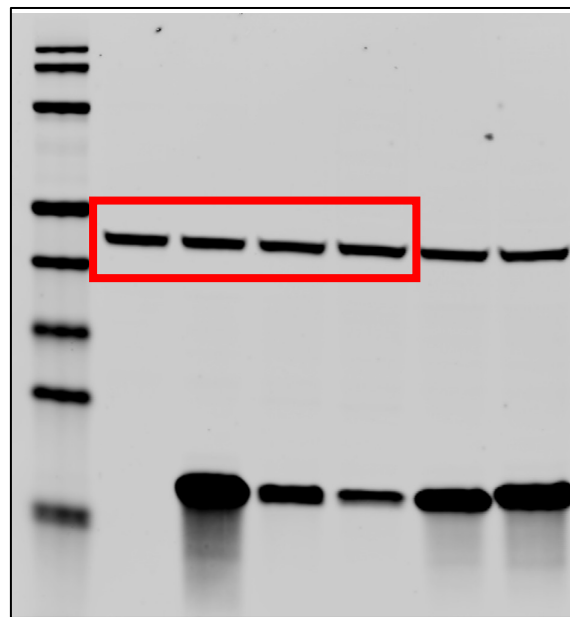

Actin

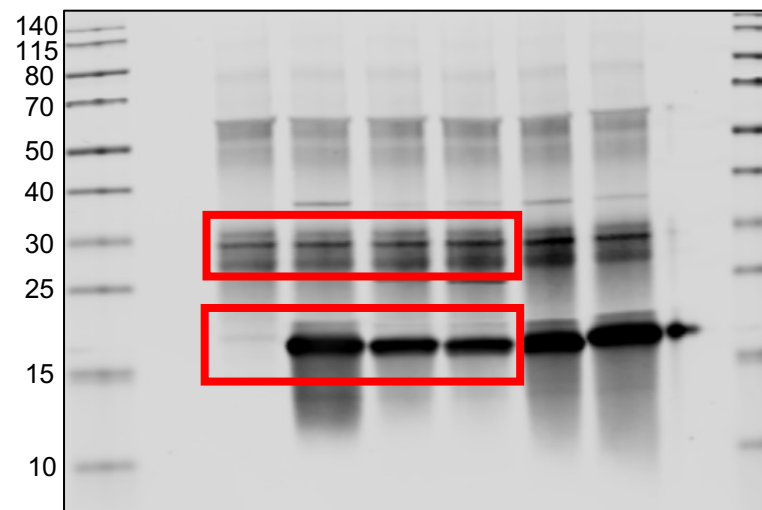

Ig Light Chain

FLAG

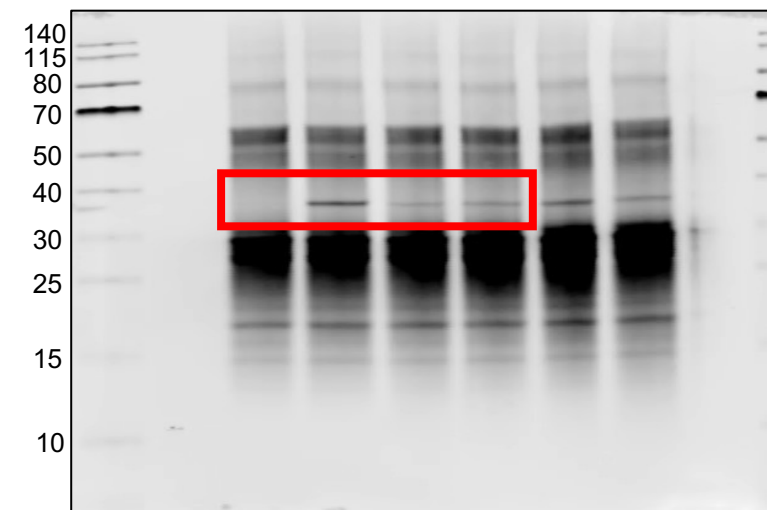

HA

Supplement: Supplementary file 4 — Source data Fig. 2 [file 44318_2024_334_MOESM4_ESM.zip › Figure 2/Figure 2E Blots.pdf]

Figure 2D

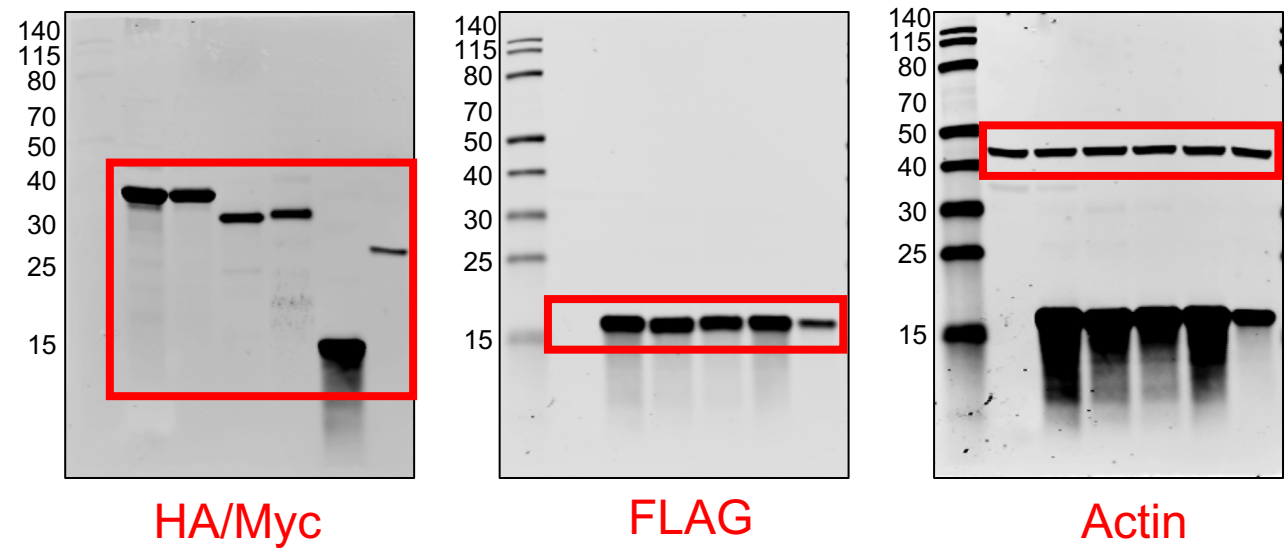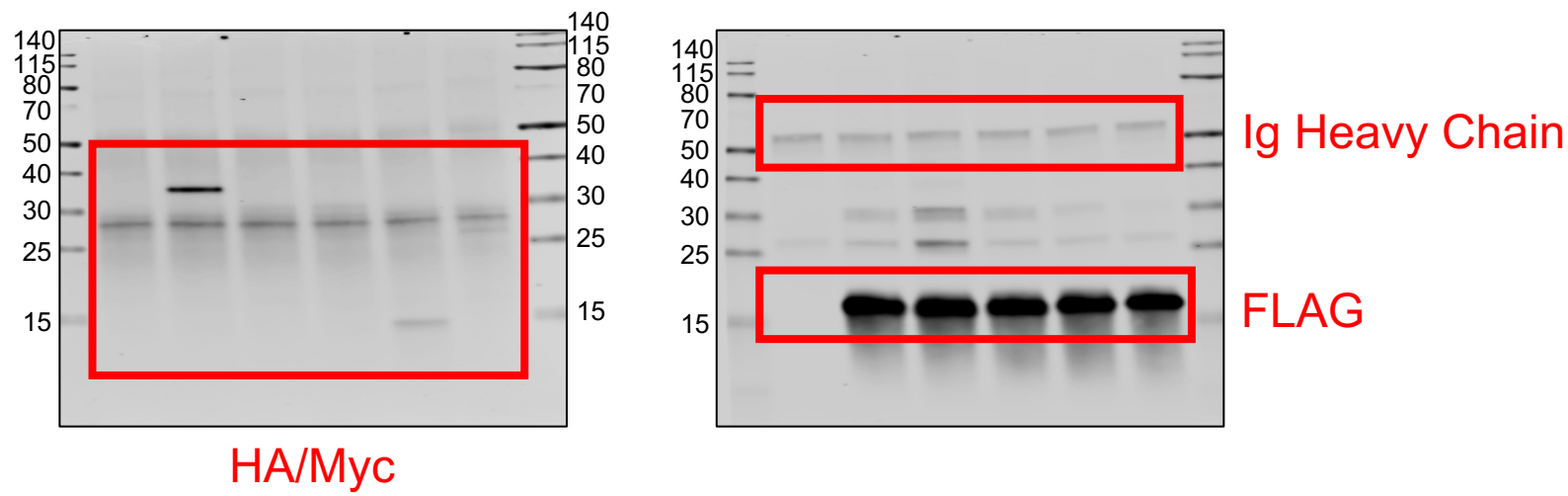

Supplement: Supplementary file 4 — Source data Fig. 2 [file 44318_2024_334_MOESM4_ESM.zip › Figure 2/Figure 2D Blots.pdf]

Figure 3B

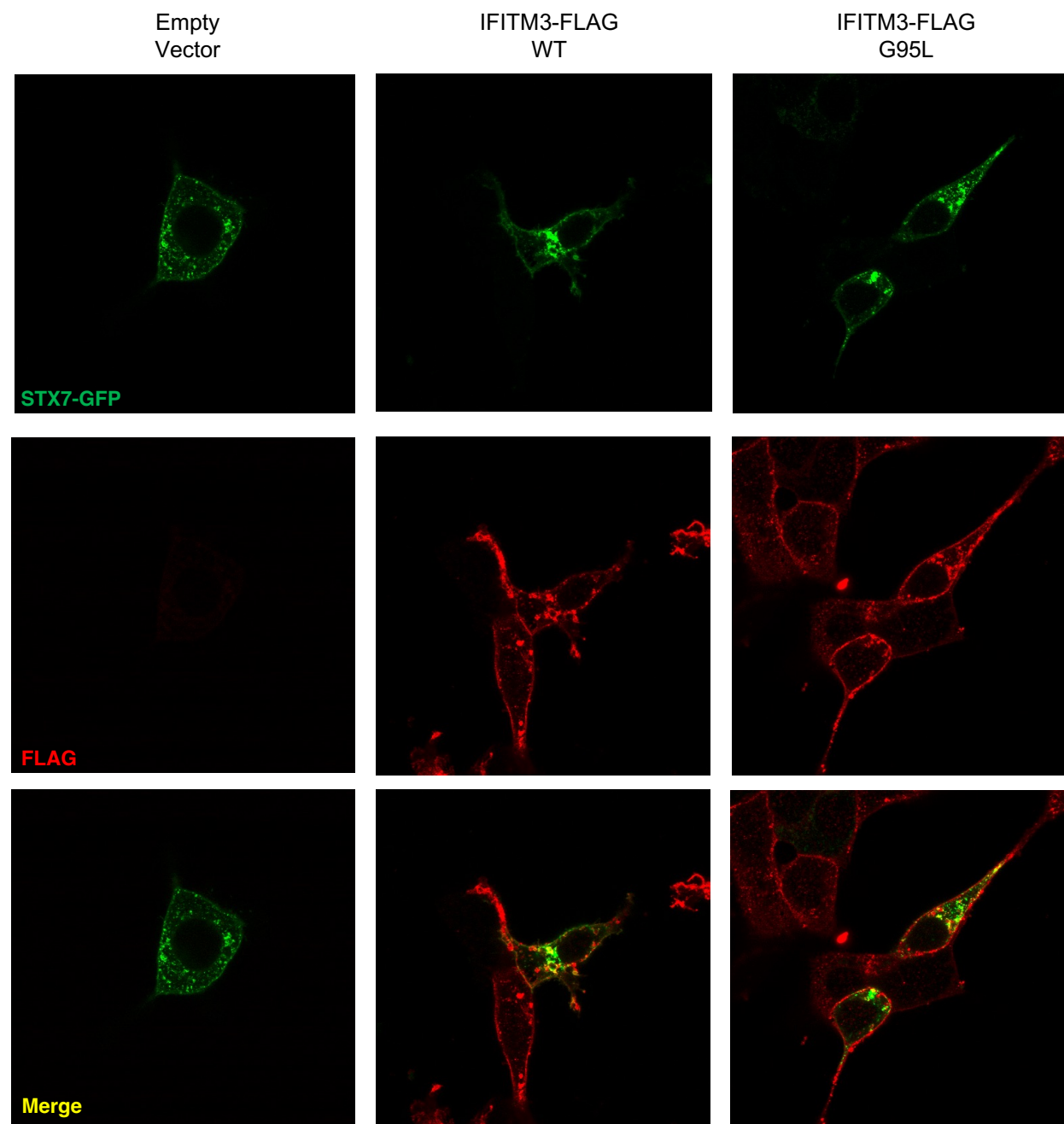

Supplement: Supplementary file 5 — Source data Fig. 3 [file 44318_2024_334_MOESM5_ESM.zip › Figure 3/Figure 3B Images.pdf]

Figure 3A

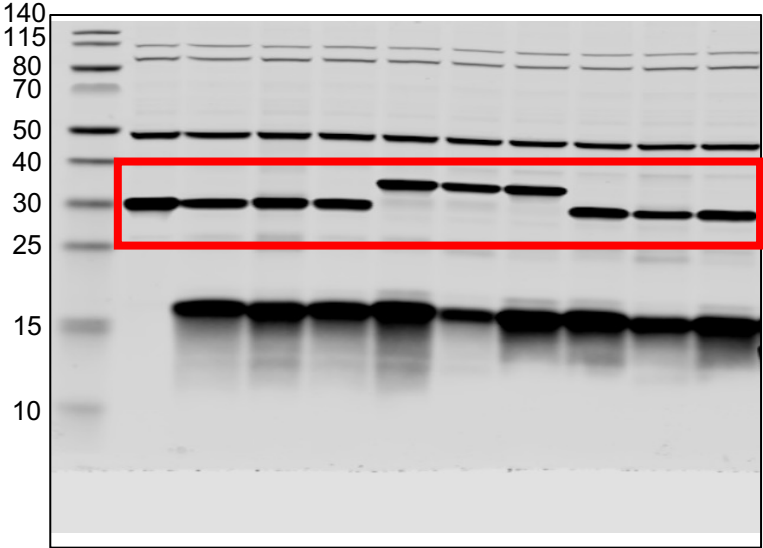

HA

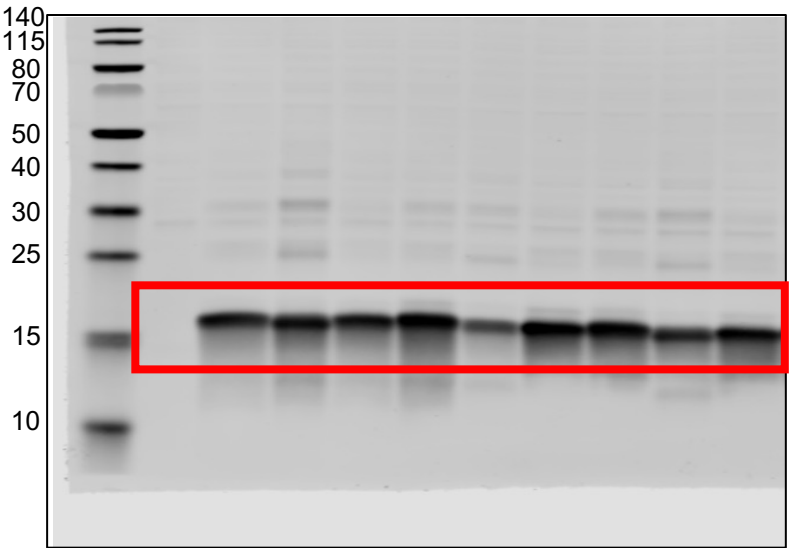

FLAG

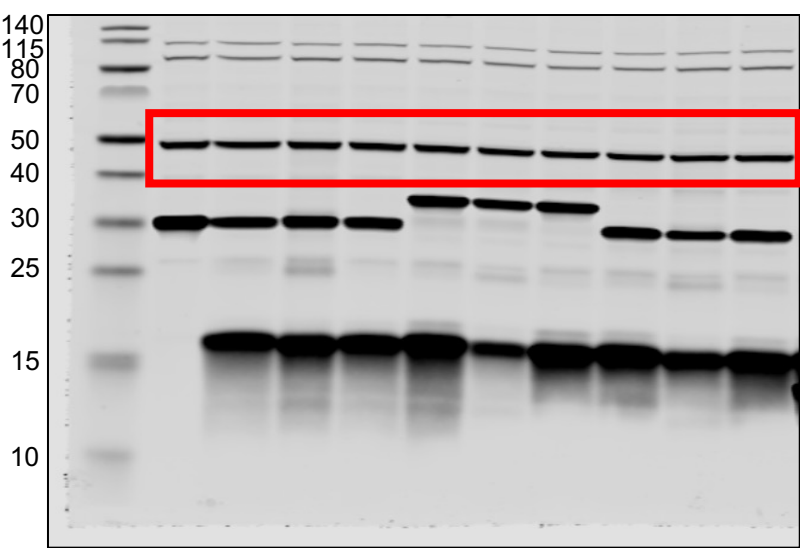

Tubulin

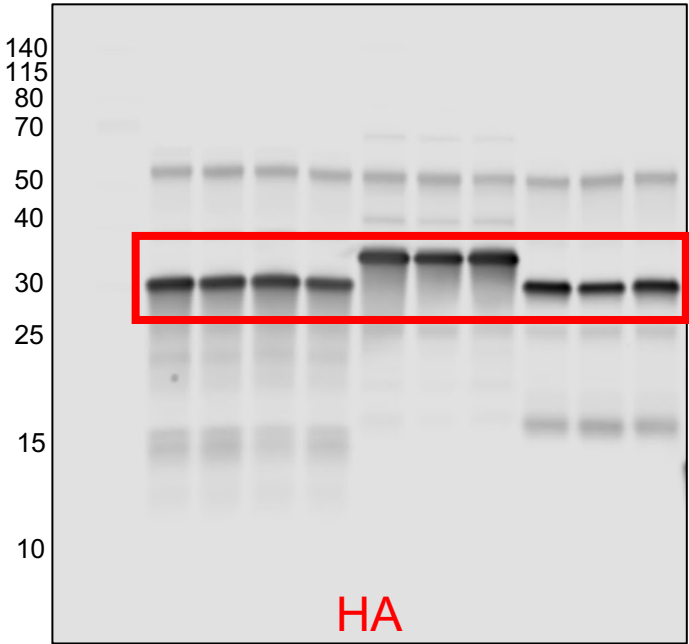

HA

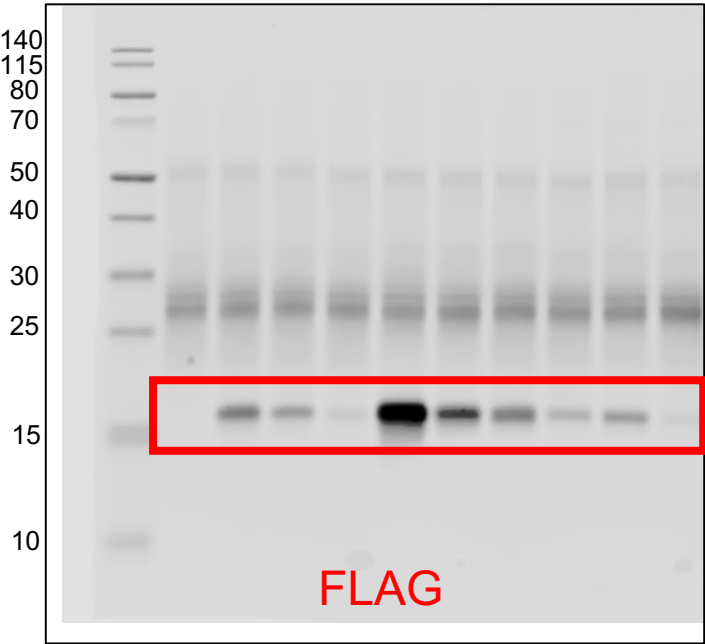

FLAG

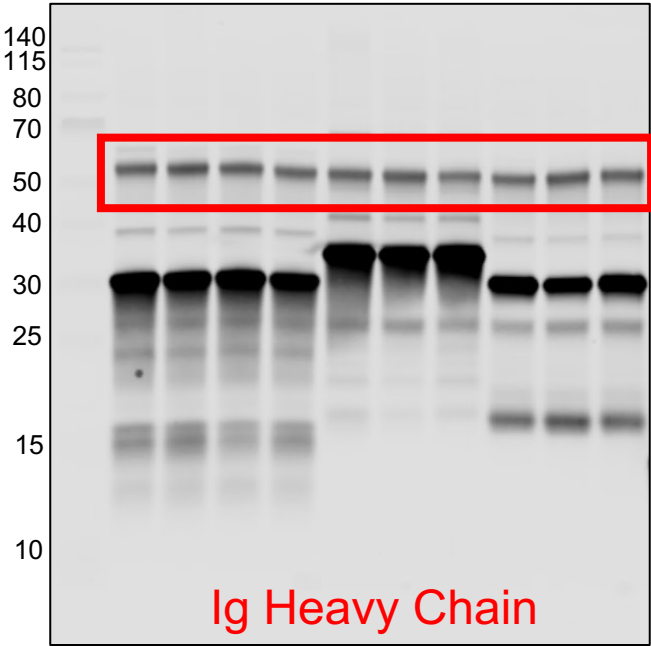

Ig Heavy Chain

Supplement: Supplementary file 5 — Source data Fig. 3 [file 44318_2024_334_MOESM5_ESM.zip › Figure 3/Figure 3A Blots.pdf]

Figure 4B

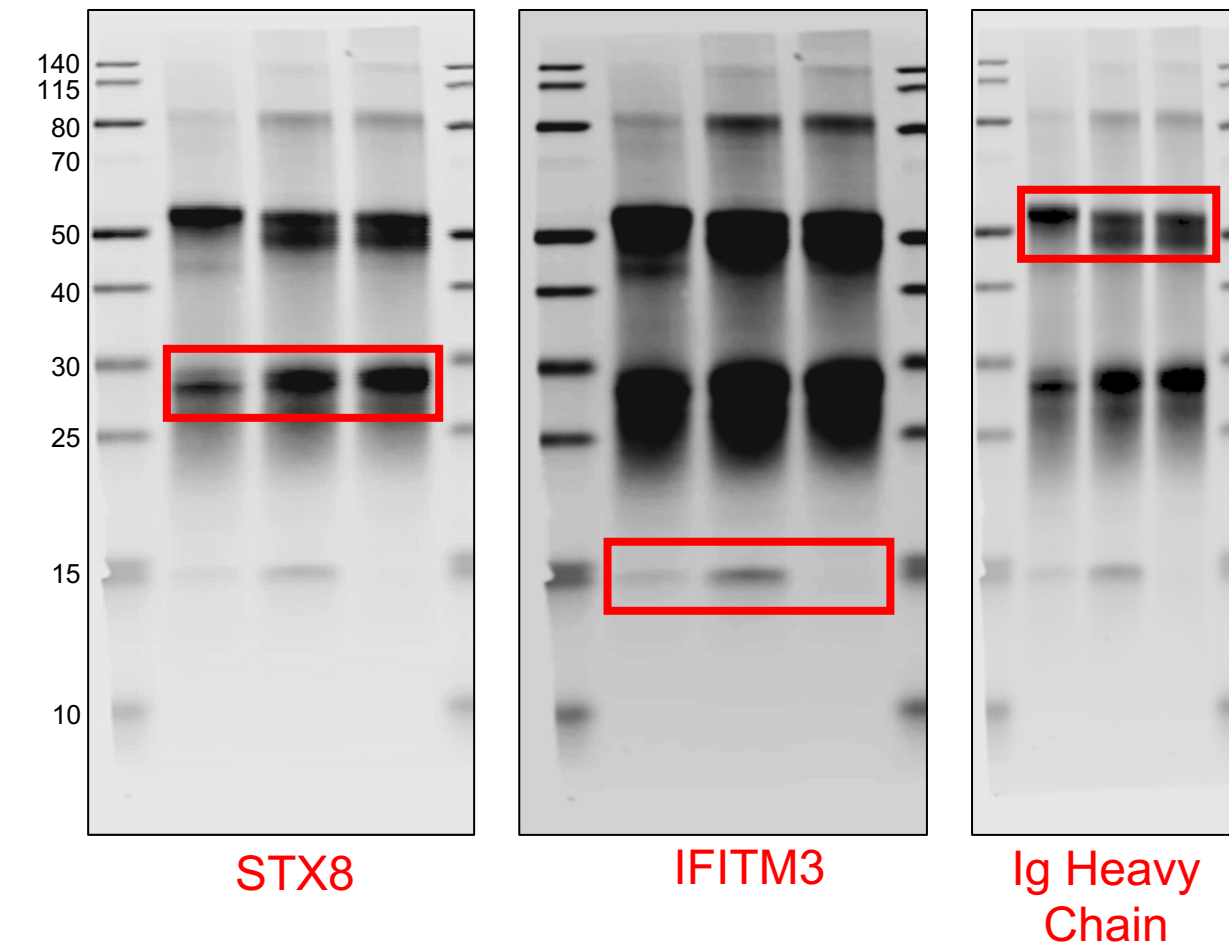

Supplement: Supplementary file 6 — Source data Fig. 4 [file 44318_2024_334_MOESM6_ESM.zip › Figure 4/Figure 4B Blots.pdf]

Figure 4D

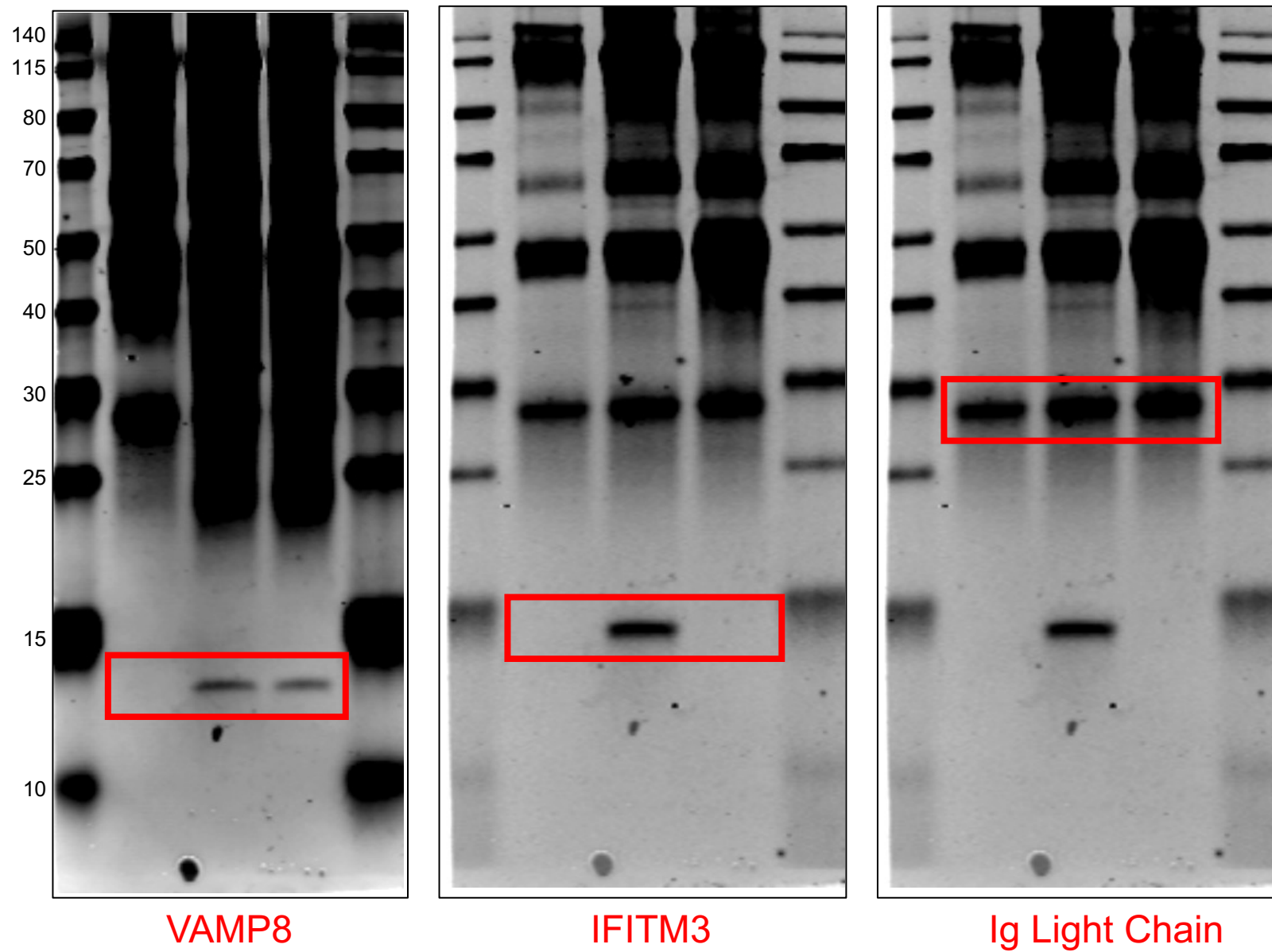

Supplement: Supplementary file 6 — Source data Fig. 4 [file 44318_2024_334_MOESM6_ESM.zip › Figure 4/Figure 4D Blots.pdf]

Figure 4C

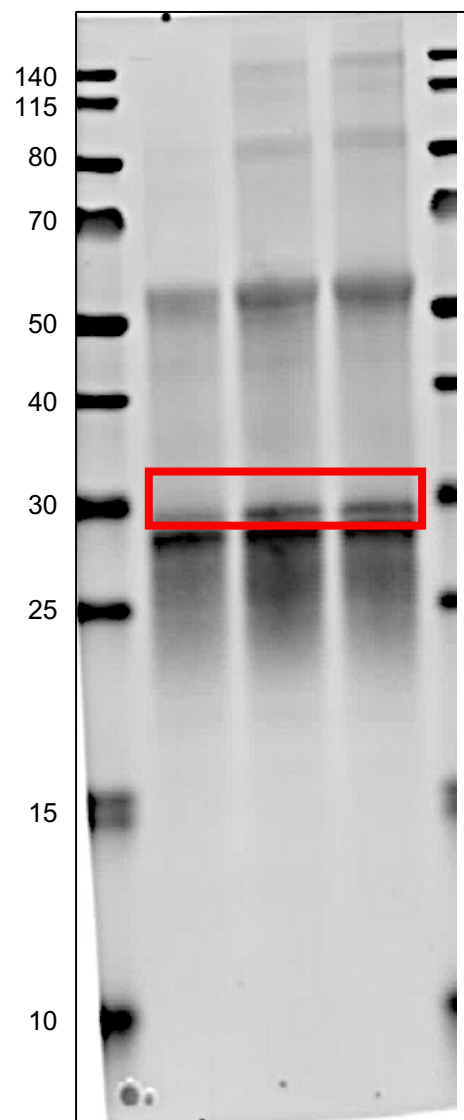

Vti1b

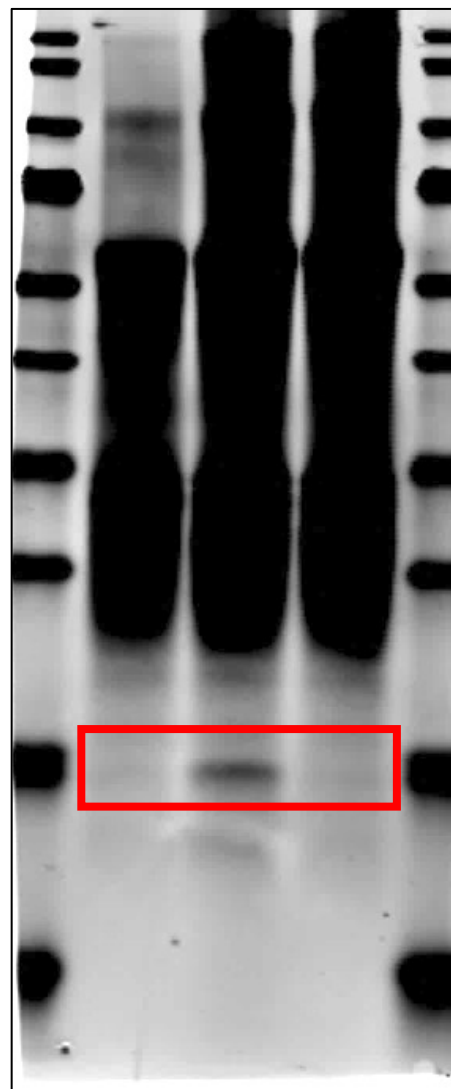

IFITM3

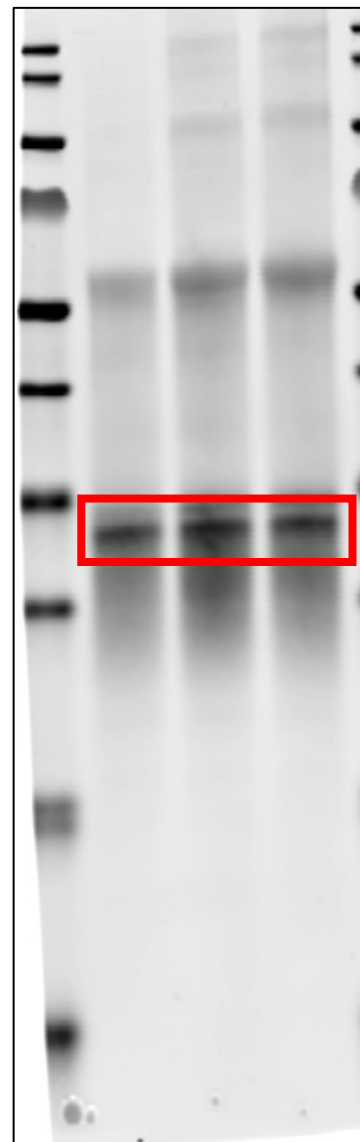

Ig Light Chain

Supplement: Supplementary file 6 — Source data Fig. 4 [file 44318_2024_334_MOESM6_ESM.zip › Figure 4/Figure 4C Blots.pdf]

Figure 4E

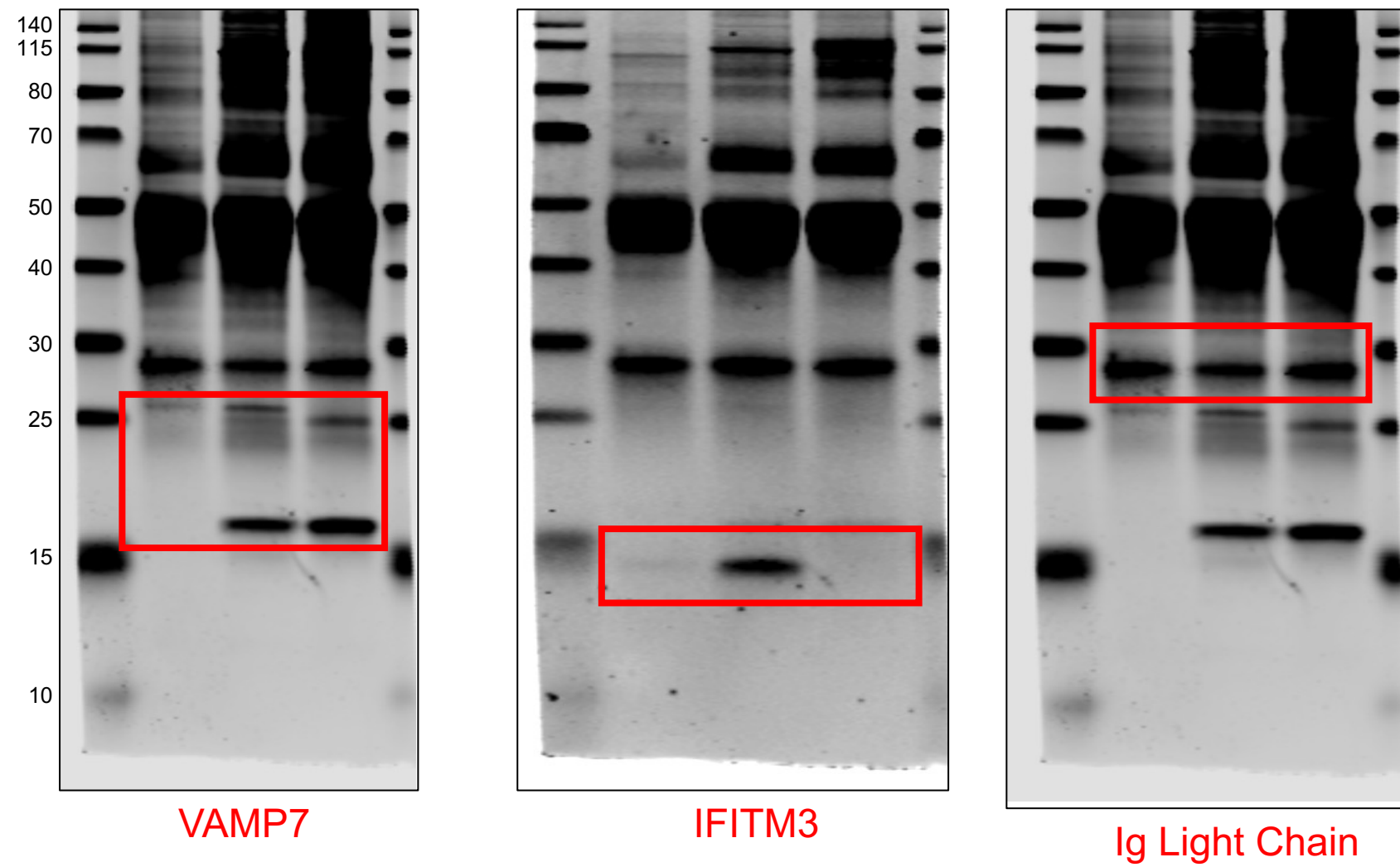

Supplement: Supplementary file 6 — Source data Fig. 4 [file 44318_2024_334_MOESM6_ESM.zip › Figure 4/Figure 4E Blots.pdf]

Figure 4F

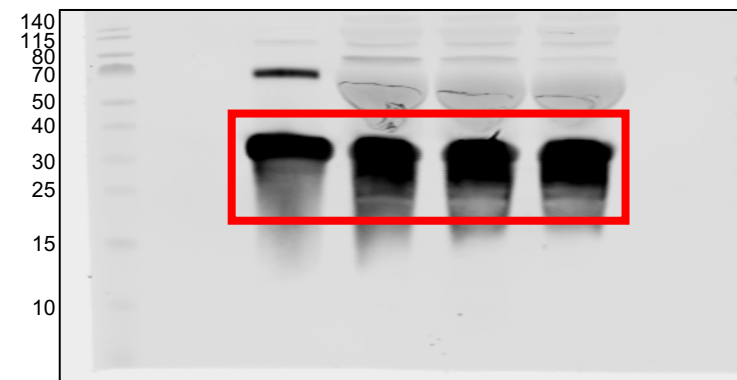

STX7

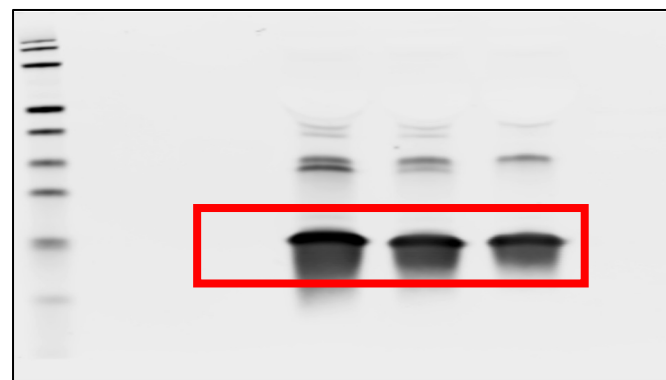

IFITM3

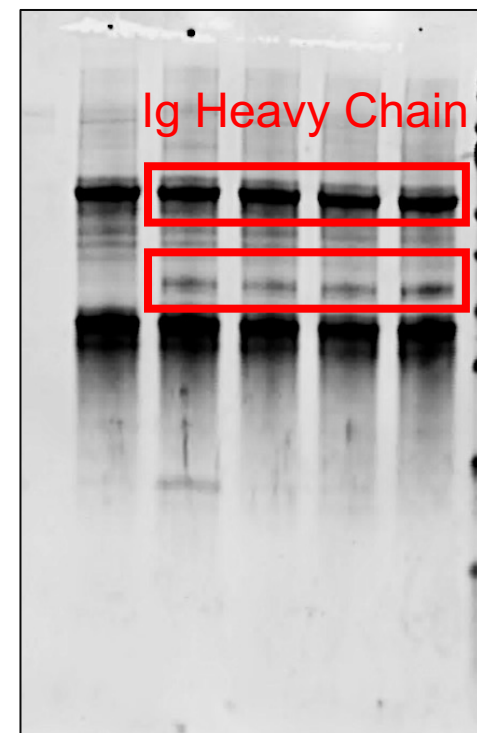

STX7

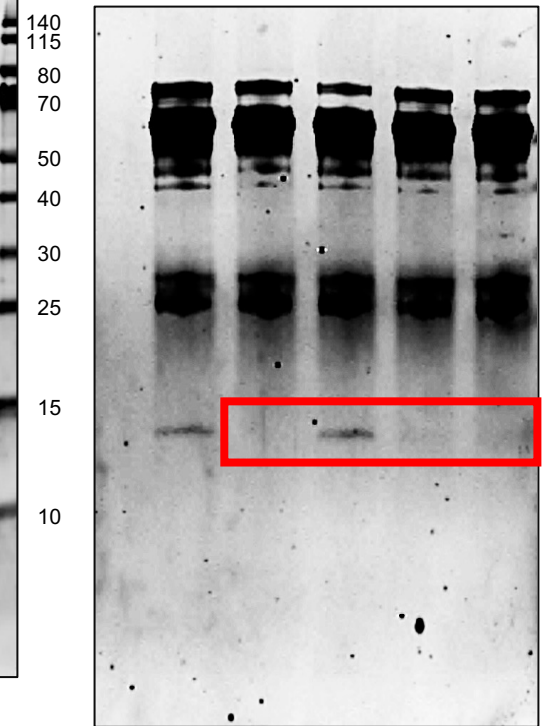

IFITM3

Supplement: Supplementary file 6 — Source data Fig. 4 [file 44318_2024_334_MOESM6_ESM.zip › Figure 4/Figure 4F Blots.pdf]

Figure 4A

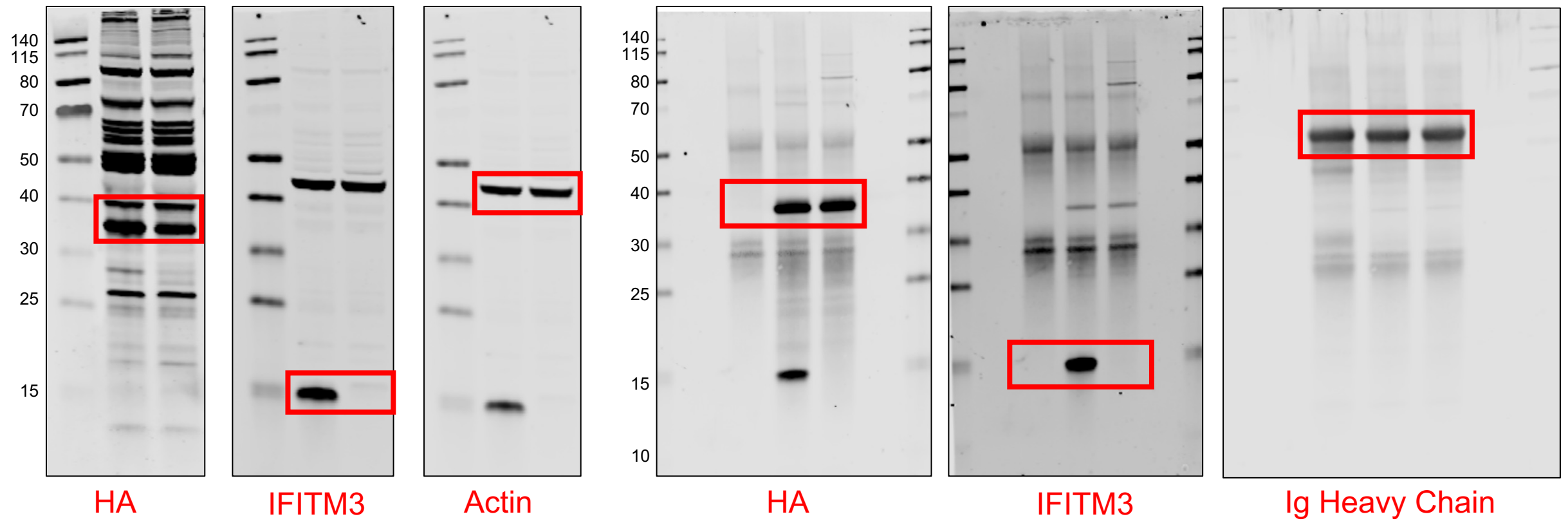

Supplement: Supplementary file 6 — Source data Fig. 4 [file 44318_2024_334_MOESM6_ESM.zip › Figure 4/Figure 4A Blots.pdf]

Figure 5B

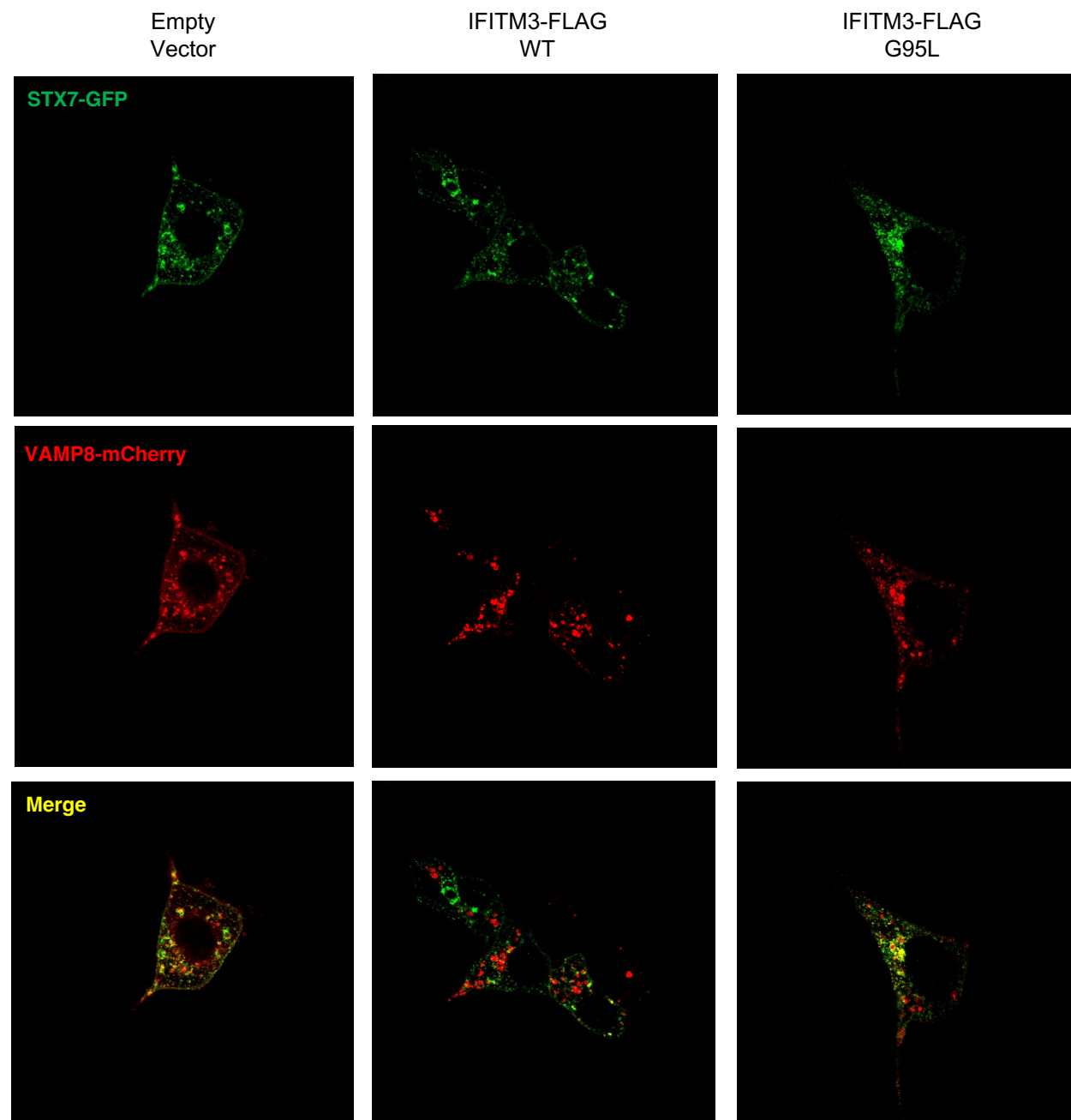

Supplement: Supplementary file 7 — Source data Fig. 5 [file 44318_2024_334_MOESM7_ESM.zip › Figure 5/Figure 5B Images.pdf]

Figure 5A

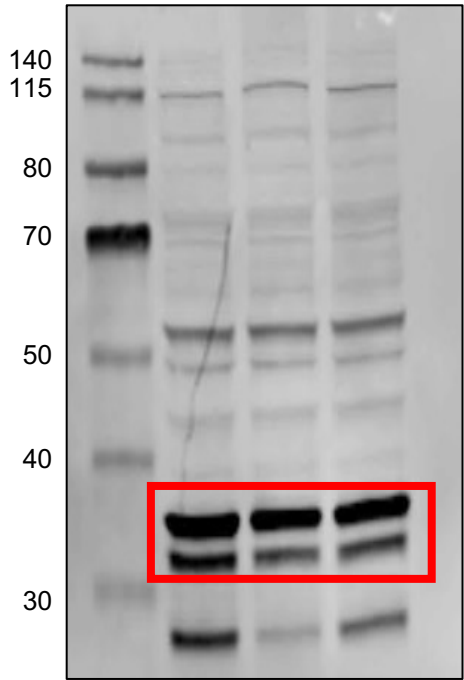

HA

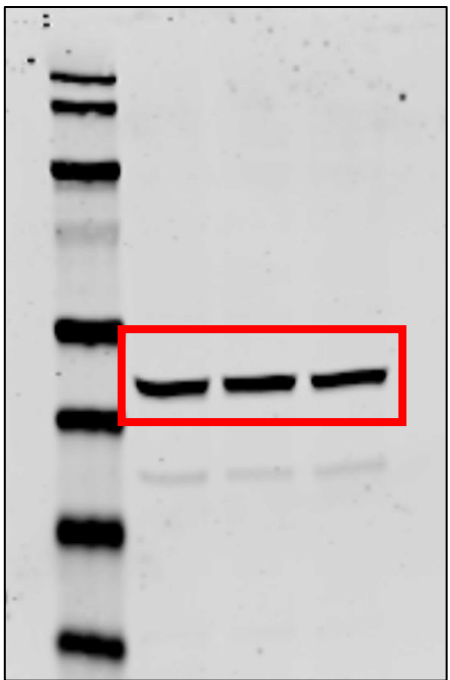

Actin

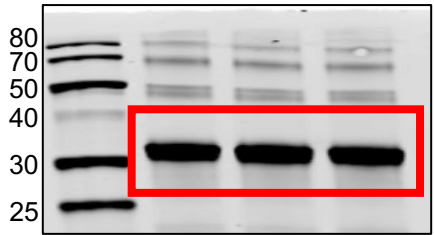

HA

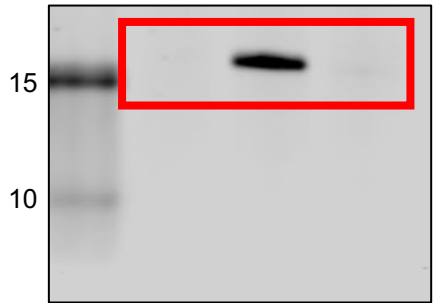

FLAG

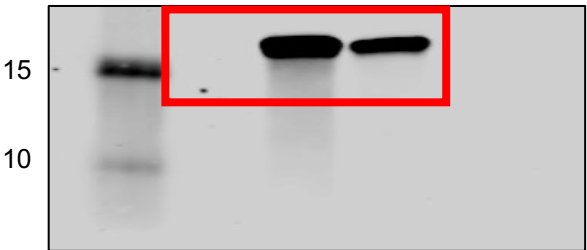

FLAG

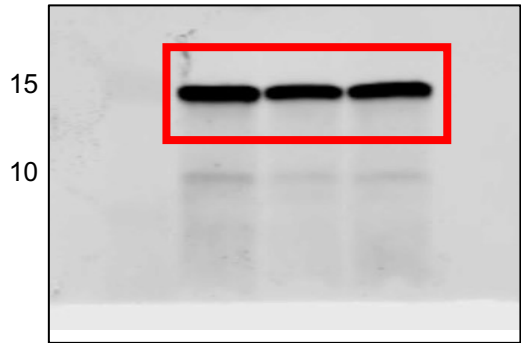

Myc

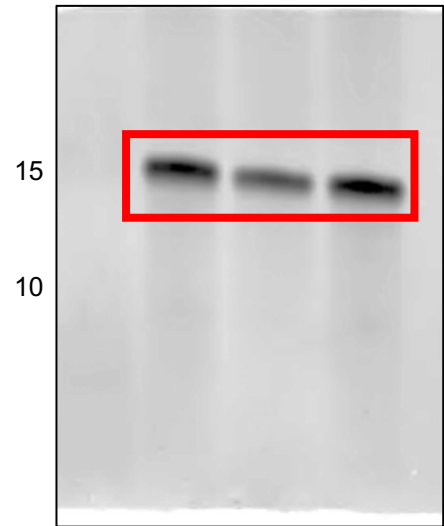

Myc

Supplement: Supplementary file 7 — Source data Fig. 5 [file 44318_2024_334_MOESM7_ESM.zip › Figure 5/Figure 5A Blots.pdf]

Figure 5D

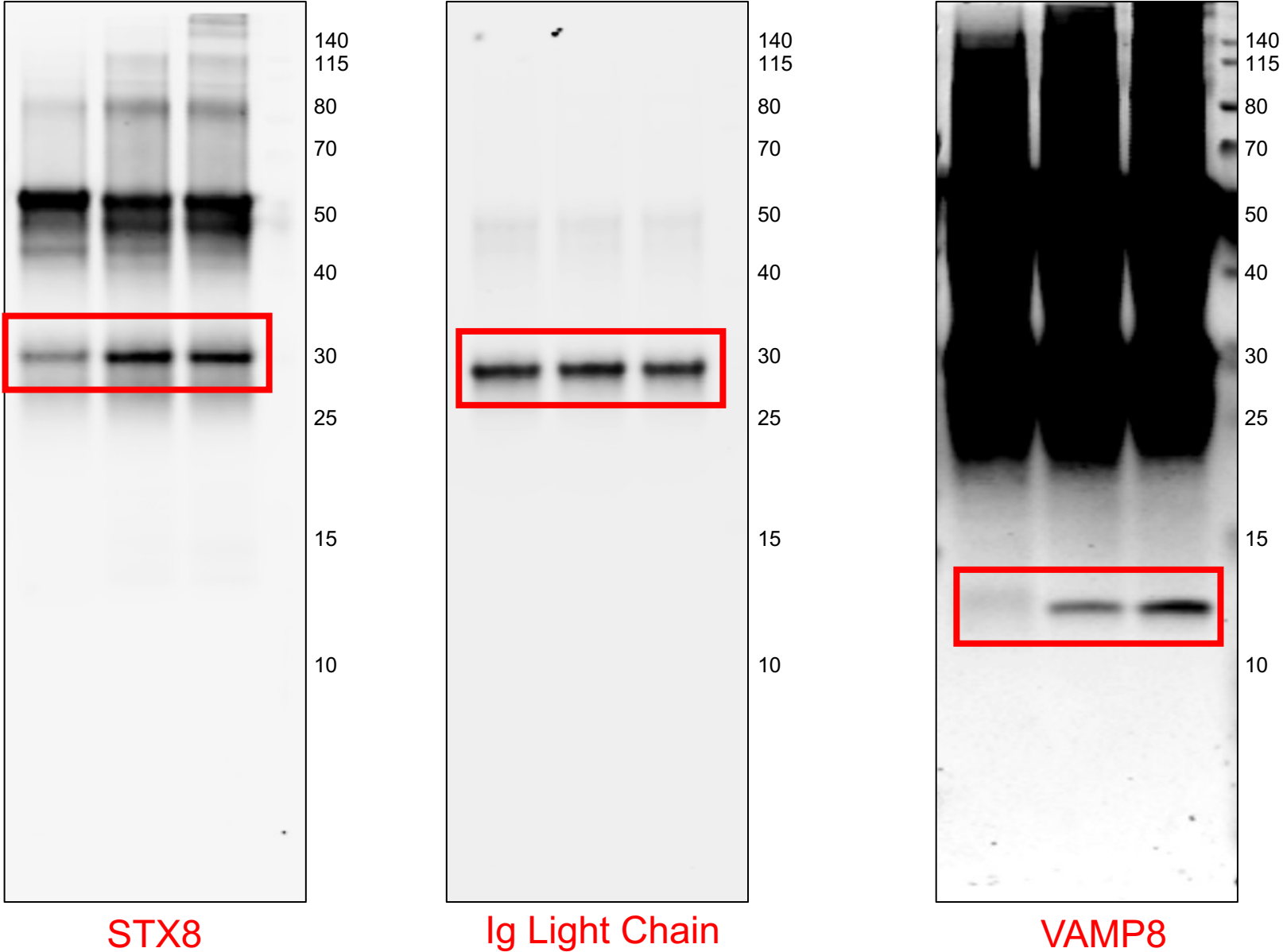

Supplement: Supplementary file 7 — Source data Fig. 5 [file 44318_2024_334_MOESM7_ESM.zip › Figure 5/Figure 5D Blots.pdf]

Figure 5C

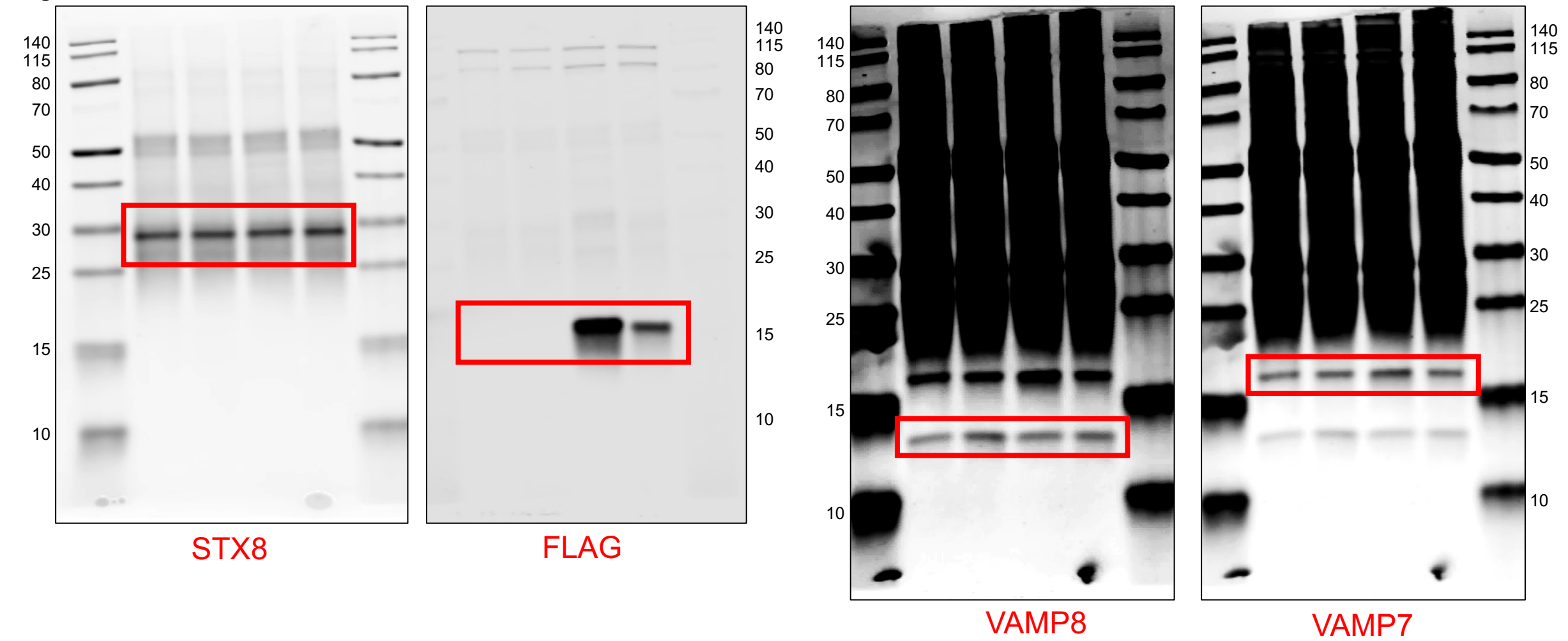

Supplement: Supplementary file 7 — Source data Fig. 5 [file 44318_2024_334_MOESM7_ESM.zip › Figure 5/Figure 5C Blots.pdf]

Figure 6B

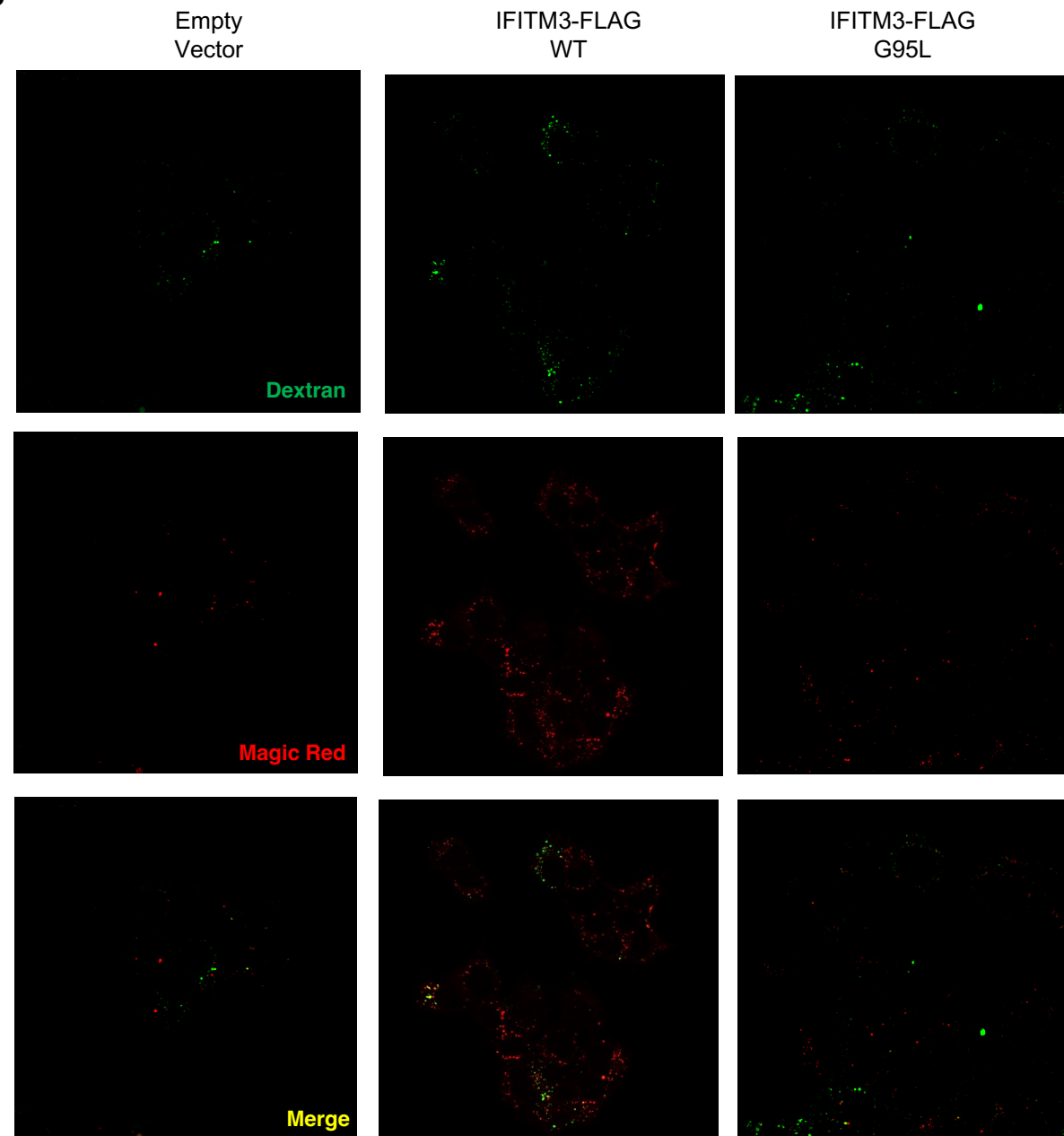

Supplement: Supplementary file 8 — Source data Fig. 6 [file 44318_2024_334_MOESM8_ESM.zip › Figure 6/Figure 6B Images.pdf]

Figure 6A

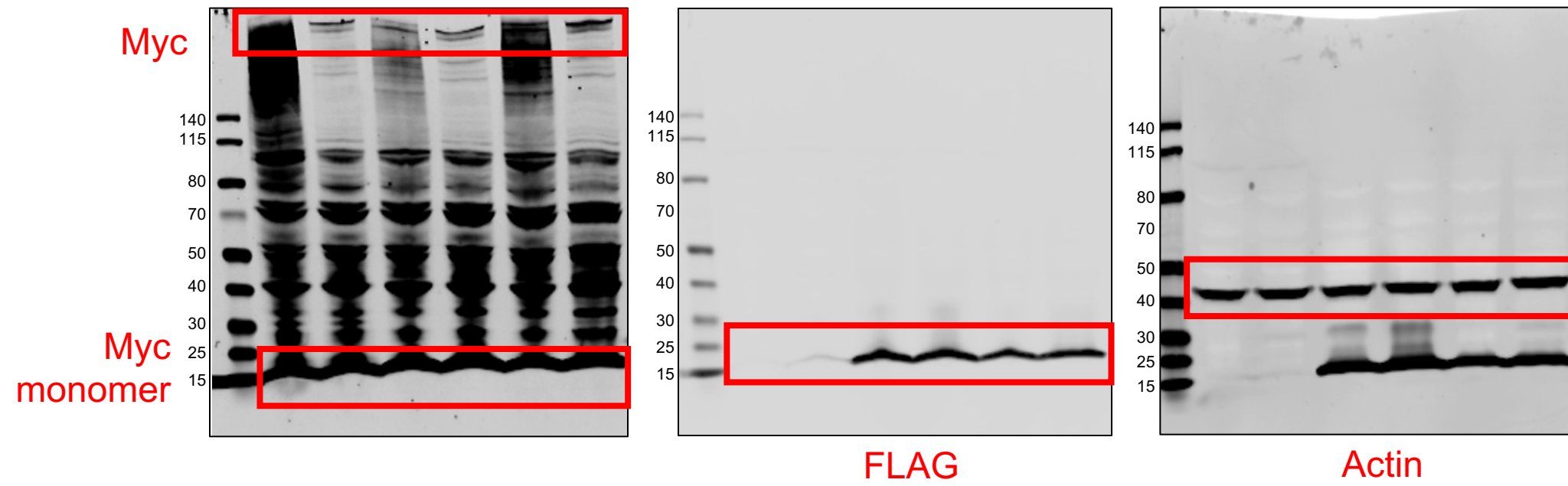

Supplement: Supplementary file 8 — Source data Fig. 6 [file 44318_2024_334_MOESM8_ESM.zip › Figure 6/Figure 6A Blots.pdf]
