# Supplementary material for: SNARE mimicry by the CD225 domain of IFITM3 enables regulation of homotypic late endosome fusion
Source: EMBO J. 2024 Dec 9;44(2):534–62. doi: 10.1038/s44318-024-00334-8 (PMC11730294; doi:10.1038/s44318-024-00334-8)
Supplement: Supplementary file 11 — Expanded View Figures [file 44318_2024_334_MOESM11_ESM.pdf]

Expanded View Figures

Figure EV1. An R-SNARE-like motif is semi-conserved among human CD225 family members.

(**A**) Human CD225 proteins were aligned and trimmed to a 53-residue stretch of sequence covering the SNARE motif. Proteins are grouped by whether they contain an arginine at the central "0" layer (R-SNARE-like) or not. Surrounding heptad repeats of hydrophobic residues are labeled from "−7" to "+8" in relation to the "0" layer, and sites highlighted in yellow correspond to hydrophobic residues. (**B**) Left: HEK293T cells were co-transfected with STX1A-HA and either PRRT2-FLAG (WT, R295Q, G305W, or A320V) or Empty Vector. SDS-PAGE and immunoblotting were performed with anti-HA and anti-FLAG in whole cell lysates. Anti-actin was used as loading control. Right: From co-transfected cells, STX1A-HA was immunoprecipitated with anti-HA followed by SDS-PAGE and immunoblotting with anti-HA and anti-FLAG. Immunoglobulin chain was used as loading control. (**C**) Left: HEK293T cells were co-transfected with STX4-HA and either TUSC5-FLAG (WT or G141W) or Empty Vector. SDS-PAGE and immunoblotting were performed with anti-HA and anti-FLAG in whole cell lysates. Anti-actin was used as loading control. Right: From co-transfected cells, TUSC5-FLAG was immunoprecipitated with anti-FLAG followed by SDS-PAGE and immunoblotting with anti-HA and anti-FLAG. Light chain immunoglobulin chain was used as loading control. The HA/FLAG ratio was calculated for the indicated lanes and shown as mean and standard error (normalized relative to WT, which was set to 100%). Differences that were statistically significant from WT as determined by student's T test are indicated by (*). Exact *p* value: $p < 0.0001$. (**D**) From co-transfected cells, STX4-HA was immunoprecipitated with anti-HA followed by SDS-PAGE and immunoblotting with anti-FLAG and anti-HA. Light chain immunoglobulin was used as loading control. Numbers and tick marks left of blots indicate position and size (in kilodaltons) of protein standard in ladder. Immunoblots were performed independently three times, and a representative example is shown (except for (**D**), which was performed twice). Ig immunoglobulin, IP immunoprecipitation, EV Empty Vector, WT wild-type. Source data are available online for this figure.

 © The Author(s)

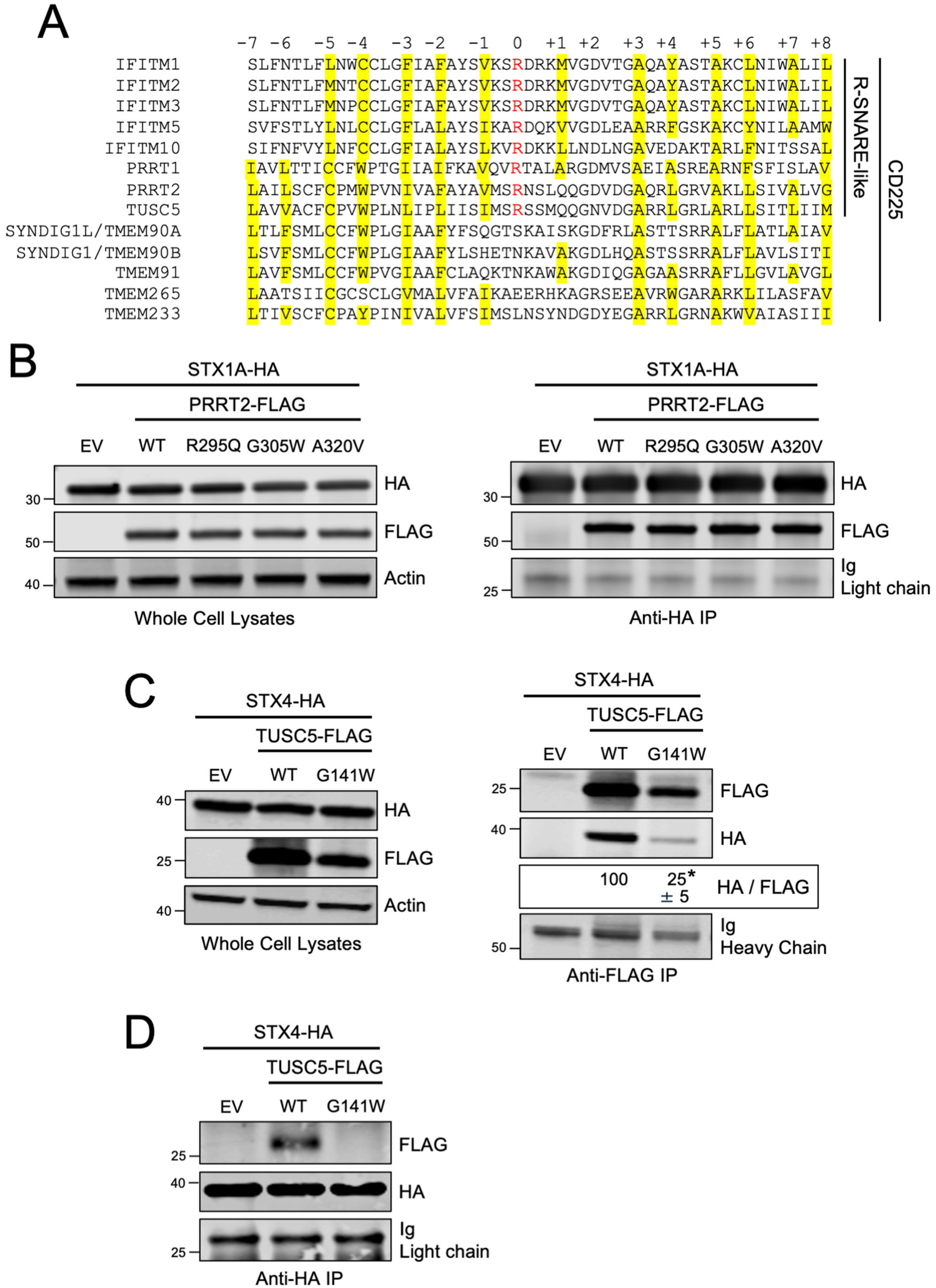
A
-7 -6 -5 -4 -3 -2 -1 0 +1 +2 +3 +4 +5 +6 +7 +8
IFITM1 SLFNTLFLNWCCLGFIAFAYSVKSRDRKMVGDVTGAQAYASTAKCLNIWALIL
IFITM2 SLFNTLFMNTCCLGFIAFAYSVKSRDRKMVGDVTGAQAYASTAKCLNIWALIL
IFITM3 SLFNTLFMNPCCLGFIAFAYSVKSRDRKMVGDVTGAQAYASTAKCLNIWALIL
IFITM5 SVFSTLYLNLCCLGFLALAYSIKARDQKVVGDLEAARRFGSKAKCYNILAAMW
IFITM10 SIFNFVYLNFCCLGFIALAYSLKVRDKKLLNDLNGAVEDAKTARLFNITSSAL
PRRT1 IAVLTTICCFWPTGIIAIFKAVQVRTALARGDMVSAEIASREARNFSFISLAV
PRRT2 LAILSCFCPMWPVNIVAFAYAVMSRNSLQQGDVDGAQRLGRVAKLLSIVALVG
TUSC5 LAVVACFCPVWPLNLIPLIISIMSRSSMQQGNVDGARRLGRLARLLSITLIIM
SYNDIG1L/TMEM90A LTLFSMLCCFWPLGIAAFYFSQGTSKAISKGDFRLASTTSRRALFLATLAIAV
SYNDIG1/TMEM90B LSVFSMLCCFWPLGIAAFYLSHETNKAVAKGDLHQASTSSRRALFLAVLSITI
TMEM91 LAVFSMLCCFWPVGIAAFCLAQKTNKAWAKGDIQGAGAASRRAFLLGVLAVGL
TMEM265 LAATSIICGCSCLGVMALVFAIKAEERHKAGRSEEAVRWGARARKLILASFAV
TMEM233 LTIVSCFCPAYPINIVALVFSIMSLNSYNDGDYEGARRLGRNAKWVAIASIII
R-SNARE-like
CD225
B
STX1A-HA
PRRT2-FLAG
EV WT R295Q G305W A320V
HA
FLAG
Actin
30
50
40
Whole Cell Lysates
STX1A-HA
PRRT2-FLAG
EV WT R295Q G305W A320V
HA
FLAG
Ig
Light chain
30
50
25
Anti-HA IP
C
STX4-HA
TUSC5-FLAG
EV WT G141W
HA
FLAG
Actin
40
25
40
Whole Cell Lysates
STX4-HA
TUSC5-FLAG
EV WT G141W
FLAG
HA
100
25*
± 5
HA / FLAG
Ig
Heavy Chain
25
40
50
Anti-FLAG IP
D
STX4-HA
TUSC5-FLAG
EV WT G141W
FLAG
HA
Ig
Light chain
25
40
25
Anti-HA IP

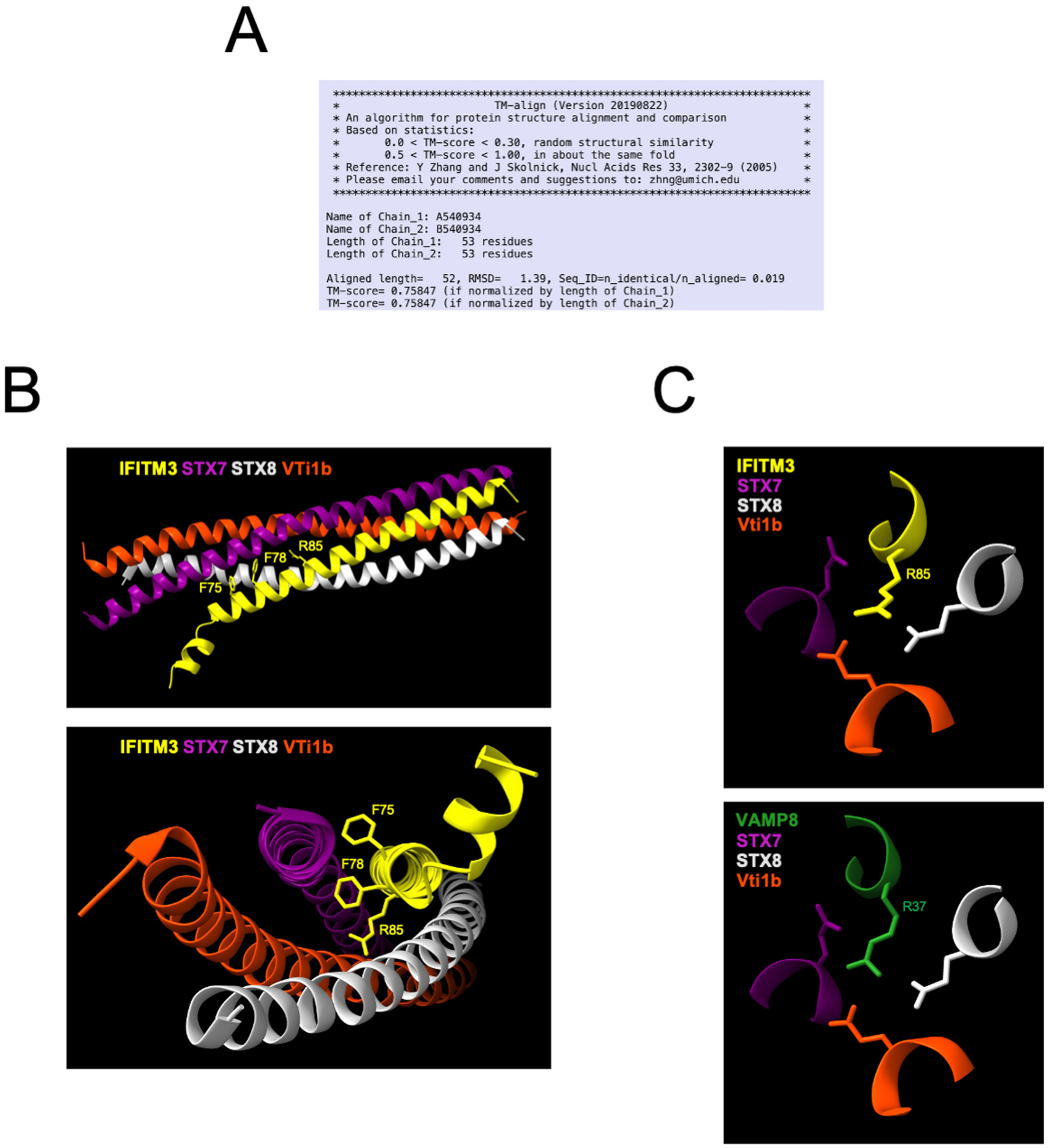

Figure EV2. IFITM3 contains a motif resembling the alpha helical R-SNARE motif of VAMP8.

(**A**) The R-SNARE-like motif of IFITM3 and the R-SNARE motif of VAMP8 (53 residues each) were compared by predictive protein structure alignment and comparison algorithm TM-align. A TM score of 0.75847 was recorded, indicating that the two regions are likely to adopt the same alpha helical protein fold. (**B**) Structural prediction of the R-SNARE-like motif of IFITM3 was performed with Alphafold and FATCAT. Residues 55-113 of IFITM3 were modeled against a template of the R-SNARE motif of VAMP8 which was previously crystallized as part of the STX7-STX8-Vti1b-VAMP8 trans-SNARE complex (PDB: 1GL2). VAMP8 was then swapped with the predicted structure of IFITM3 and shown in relation to the coiled-coiled structure formed with STX7, STX8, and Vti1b. Top: the side chains of residues F75, F78, and R85 of IFITM3 are depicted in yellow and labeled. Bottom: alternative view of the side chains of F75, F78, and R85 of IFITM3. (**C**) Top: examination of the central polar "0" layer of the Q-SNAREs STX7, STX8, Vti1b in relation to the R85 residue in the predicted configuration of IFITM3. Bottom: examination of the central polar "0" layer of the STX7-STX8-Vti1b-VAMP8 complex (PDB: IGL2). Source data are available online for this figure.

 © The Author(s)

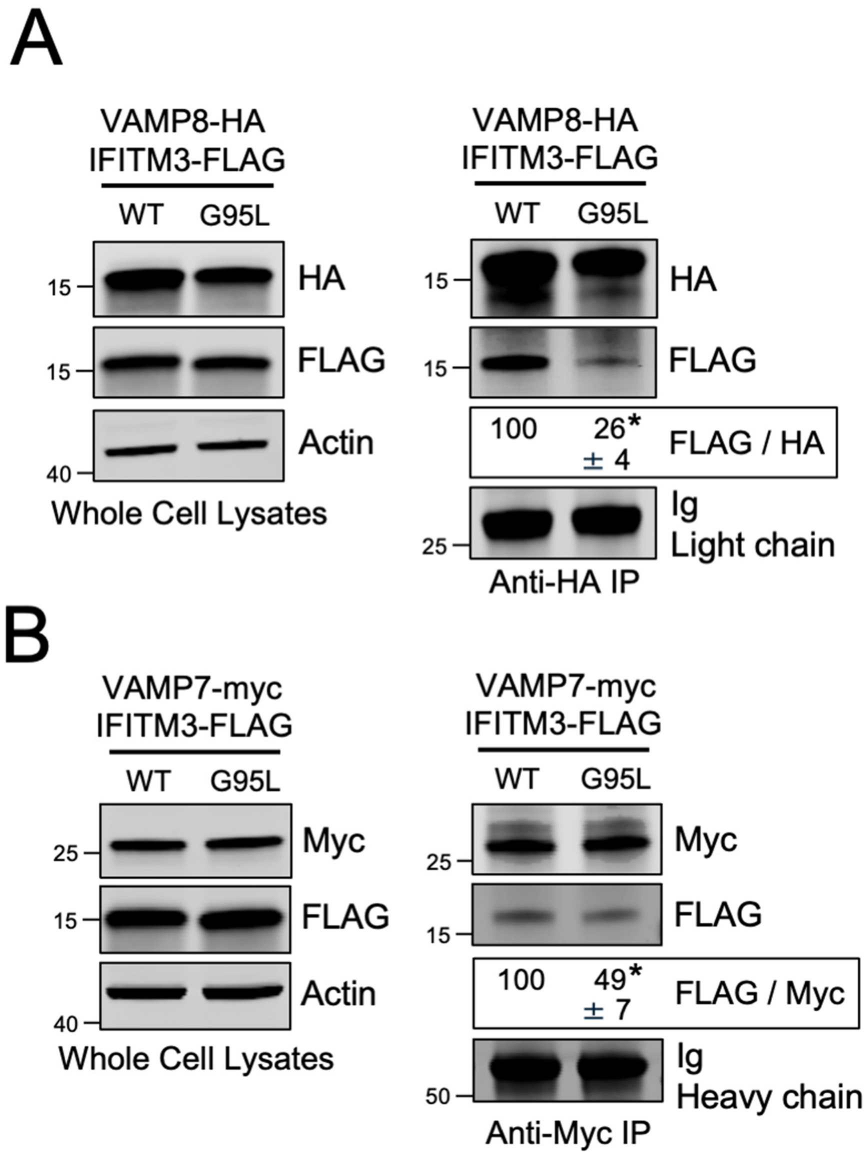

Figure EV3. IFITM3 interacts with the R-SNAREs VAMP8 and VAMP7.

(**A**) Left: HEK293T cells were co-transfected with VAMP8-HA and IFITM3-FLAG (WT or G95L). SDS-PAGE and immunoblotting were performed with anti-HA and anti-FLAG in whole cell lysates. Anti-actin was used as loading control. Right: From co-transfected cells, VAMP8-HA was immunoprecipitated with anti-HA followed by SDS-PAGE and immunoblotting with anti-HA and anti-FLAG. Light chain immunoglobulin chain was used as loading control. The FLAG/HA ratio was calculated for the indicated lanes and shown as mean and standard error (normalized relative to WT, which was set to 100%). Differences that were statistically significant from WT as determined by student's T test are indicated by (*). Exact *p* value: $p < 0.0001$. (**B**) Left: HEK293T cells were co-transfected with VAMP7-myc and IFITM3-FLAG (WT or G95L). SDS-PAGE and immunoblotting were performed with anti-myc and anti-FLAG in whole cell lysates. Anti-actin was used as loading control. Right: From co-transfected cells, VAMP7-myc was immunoprecipitated with anti-myc followed by SDS-PAGE and immunoblotting with anti-myc and anti-FLAG. Light chain immunoglobulin chain was used as loading control. The FLAG/Myc ratio was calculated for the indicated lanes and shown as mean and standard error (normalized relative to WT, which was set to 100%). Differences that were statistically significant from WT as determined by student's T test are indicated by (*). Exact *p* value: $p = 0.0023$. Numbers and tick marks left of blots indicate position and size (in kilodaltons) of protein standard in ladder. Immunoblots were performed independently three times, and a representative example is shown. Ig immunoglobulin, IP immunoprecipitation, WT wild-type. Source data are available online for this figure.

© The Author(s)

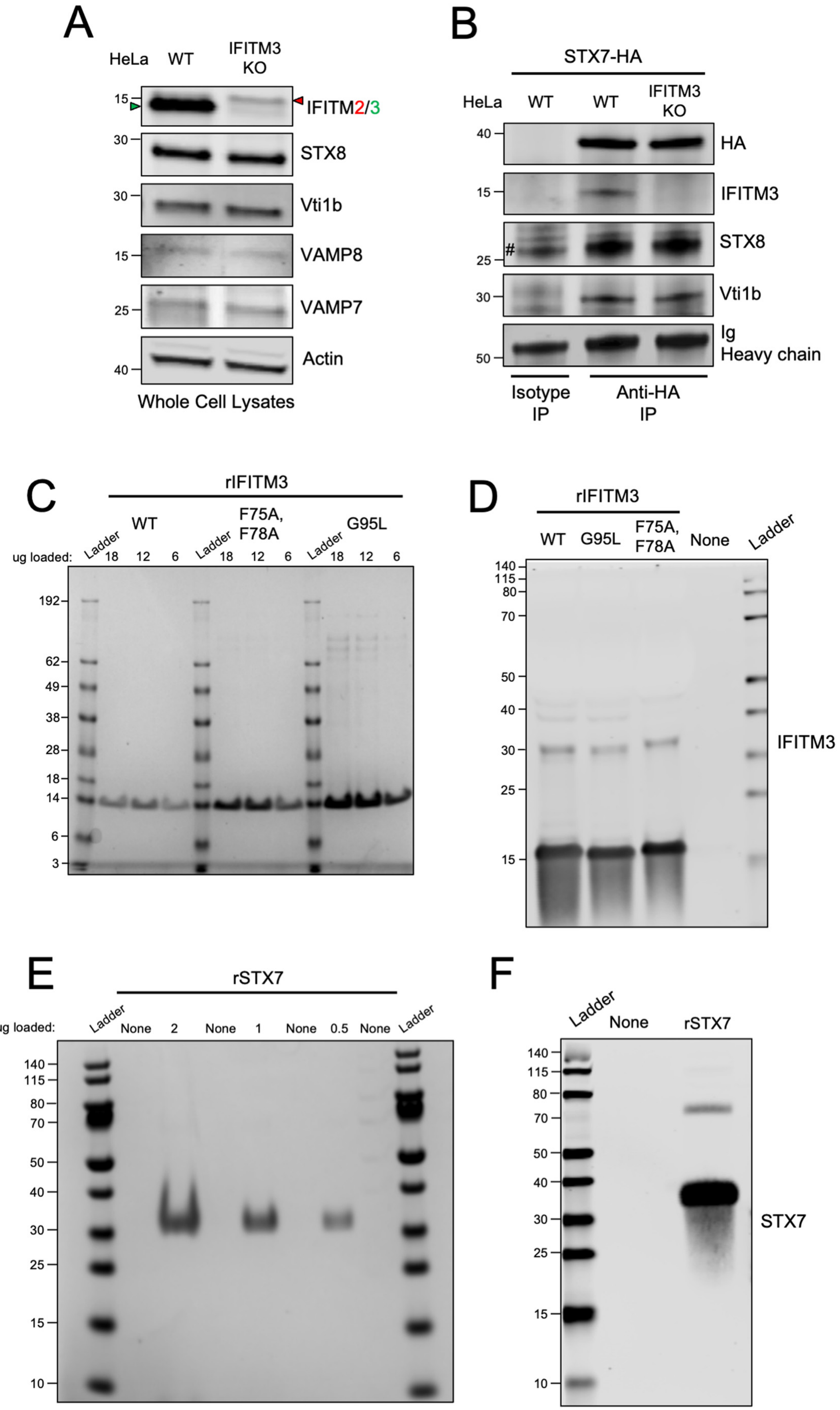
A
HeLa WT IFITM3 KO
IFITM2/3
STX8
Vti1b
VAMP8
VAMP7
Actin
Whole Cell Lysates
B
STX7-HA
HeLa WT WT IFITM3 KO
HA
IFITM3
STX8
Vti1b
Ig Heavy chain
Isotype IP
Anti-HA IP
C
rIFITM3
WT
F75A, F78A
G95L
ug loaded:
Ladder
D
rIFITM3
WT G95L F75A, F78A None Ladder
IFITM3
E
rSTX7
ug loaded:
Ladder None 2 None 1 None 0.5 None Ladder
F
Ladder None rSTX7
STX7

◀ **Figure EV4. IFITM3 does not impair the STX7/STX8 or STX7/Vti1b interactions.**

(**A**) HeLa cells (WT or *IFITM3* KO) were subjected to whole cell lysis, SDS-PAGE, and immunoblotting with anti-IFITM2/3, anti-STX8, anti-Vti1b, anti-VAMP8, anti-VAMP7, and anti-actin. Red and green arrows indicate IFITM3 and IFITM2, respectively, recognized by the anti-IFITM2/3 antibody. (**B**) STX7-HA was transfected into HeLa (WT or *IFITM3* KO) and immunoprecipitated with anti-HA followed by SDS-PAGE and immunoblotting with anti-HA, anti-IFITM3, anti-STX8, and anti-Vti1b. Heavy chain immunoglobulin was used as loading control. An isotype matched antibody was used as a control for immunoprecipitation. The (#) symbol denotes the presence of light chain immunoglobulin. (**C**) Purified recombinant IFITM3 protein (WT, F75/78A, or G95L) of varying inputs (6, 12, or 18 µg) were subjected to SDS-PAGE and visualized by Coomassie stain. (**D**) 20 ng of purified recombinant IFITM3 protein (WT, F75/78A, or G95L) were subjected to SDS-PAGE and immunoblotting with anti-IFITM3. (**E**) Recombinant STX7 of varying inputs (0.5, 1, or 2 µg) were subjected to SDS-PAGE and visualized by Coomassie stain. (**F**) Recombinant STX7 was subjected to SDS-PAGE and immunoblotting with anti-STX7. Numbers and tick marks left of blots indicate position and size (in kilodaltons) of protein standard in ladder. Immunoblots were performed independently two times and a representative example is shown (for (**A**) and (**B**)) or once (for (**C**), (**D**), (**E**), and (**F**)). Ig immunoglobulin, IP immunoprecipitation, EV Empty Vector, WT wild-type, r recombinant. Source data are available online for this figure.

© The Author(s)

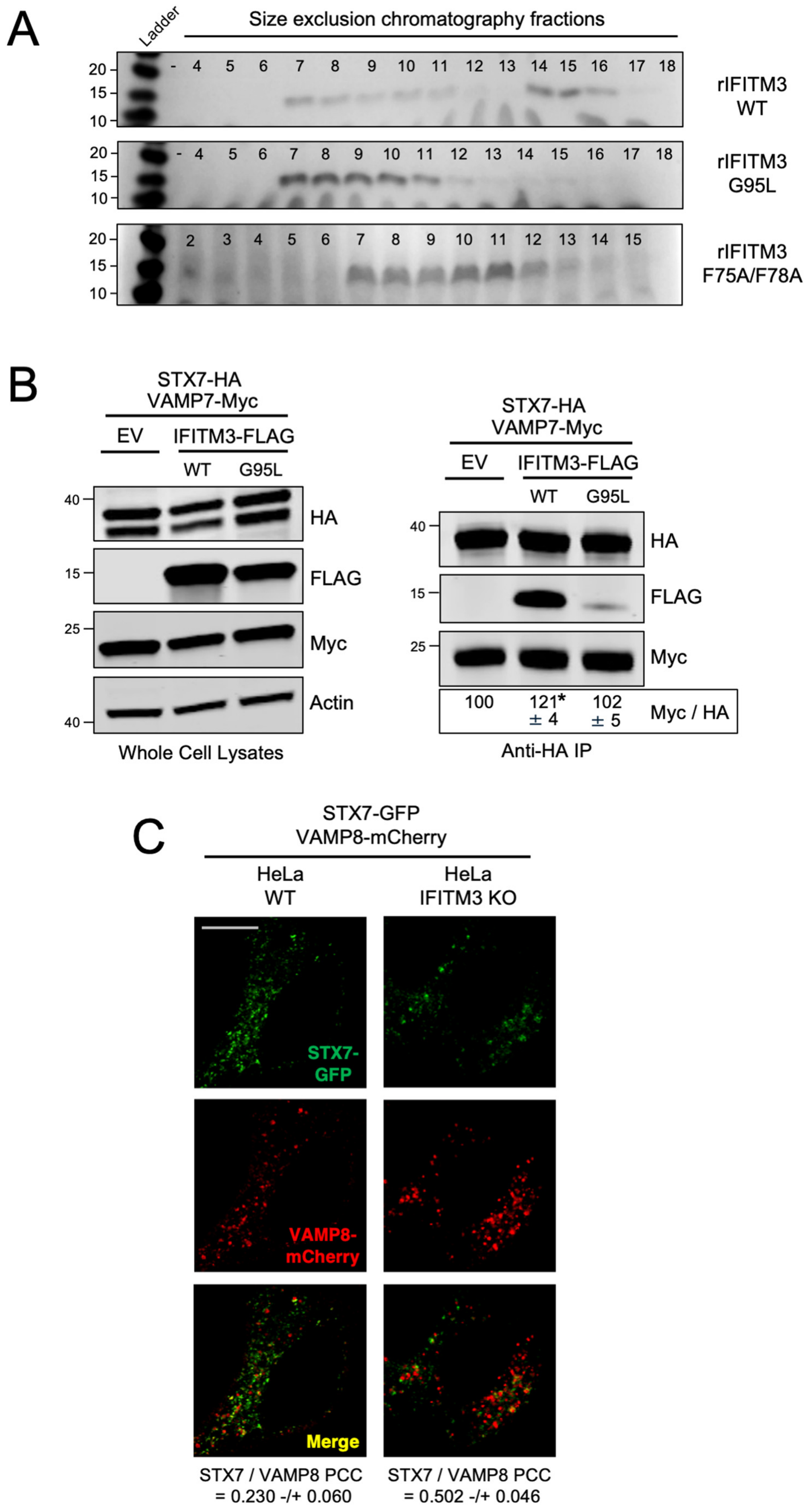
A
Ladder
Size exclusion chromatography fractions
20
15
10
- 4 5 6 7 8 9 10 11 12 13 14 15 16 17 18
rIFITM3
WT
- 4 5 6 7 8 9 10 11 12 13 14 15 16 17 18
rIFITM3
G95L
2 3 4 5 6 7 8 9 10 11 12 13 14 15
rIFITM3
F75A/F78A
B
STX7-HA
VAMP7-Myc
EV
IFITM3-FLAG
WT G95L
40
HA
15
FLAG
25
Myc
Actin
40
Whole Cell Lysates
HA
FLAG
Myc
100
121*
± 4
102
± 5
Myc / HA
Anti-HA IP
C
STX7-GFP
VAMP8-mCherry
HeLa
WT
HeLa
IFITM3 KO
STX7-
GFP
VAMP8-
mCherry
Merge
STX7 / VAMP8 PCC
= 0.230 -/+ 0.060
STX7 / VAMP8 PCC
= 0.502 -/+ 0.046

Figure EV5. IFITM3 does not impair the STX7/VAMP7 interaction.

(**A**) Size exclusion chromatography was performed on recombinant IFITM3 proteins (WT, F75/78A, and G95L) and eluted fractions were analyzed by SDS-PAGE and Coomassie staining. 150 µg recombinant protein was loaded as input and fraction numbers correspond to mL volumes eluted from column. (**B**) Left: HEK293T cells stably expressing Empty Vector, IFITM3 WT-FLAG, or IFITM3 G95L-FLAG were co-transfected with STX7-HA and VAMP7-Myc. Whole cell lysates were subjected to SDS-PAGE and immunoblotting with anti-HA, anti-FLAG, and anti-Myc. Actin was used as a loading control. Right: STX7-HA was immunoprecipitated with anti-HA followed by SDS-PAGE and immunoblotting with anti-HA, anti-FLAG, and anti-Myc. The Myc/HA ratio was calculated for the indicated lanes and shown as mean and standard error (normalized relative to Empty Vector, which was set to 100%). Differences that were statistically significant from Empty Vector as determined by one-way ANOVA are indicated by (*). Exact p values are as follows (from left to right): $p = 0.0099$, $p = 0.8782$. Numbers and tick marks left of blots indicate position and size (in kilodaltons) of protein standard in ladder. Immunoblots were performed independently three times, and a representative example is shown (except (**A**), which was performed once). (**C**) HeLa (WT or *IFITM3* KO) were transfected with STX7-GFP and VAMP8-mCherry and confocal immunofluorescence microscopy was performed. Colocalization between STX7-GFP and VAMP8-mCherry was measured by calculating the Pearson's correlation coefficient using Fiji software. Coefficients were calculated from medial Z-slices from three fields of view containing 5–15 cells per condition and presented as means and standard error. Scale bar = 15 microns. IP immunoprecipitation, WT wild-type, r recombinant. Source data are available online for this figure.

© The Author(s)
