# Supplementary material for: SNARE mimicry by the CD225 domain of IFITM3 enables regulation of homotypic late endosome fusion
Source: EMBO J. 2024 Dec 9;44(2):534–62. doi: 10.1038/s44318-024-00334-8 (PMC11730294; doi:10.1038/s44318-024-00334-8)
Supplement: Supplementary file 10 — EV and Appendix Figure Source Data [file 44318_2024_334_MOESM10_ESM.zip › EV Figure Source Data.zip!/Expanded_View_Figure_5_Source_Data.zip!/Expanded View Figure 5/Expanded View Figure 5B Blots.pdf]

Expanded View Figure 5B

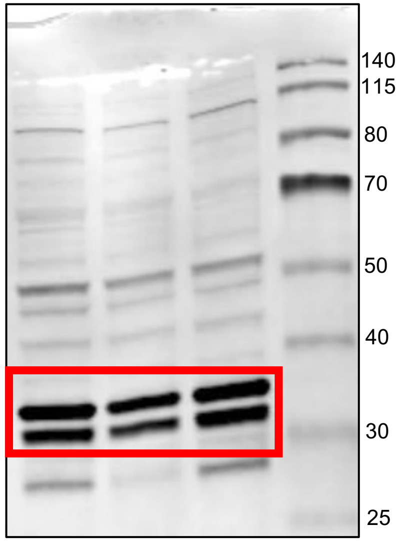

HA

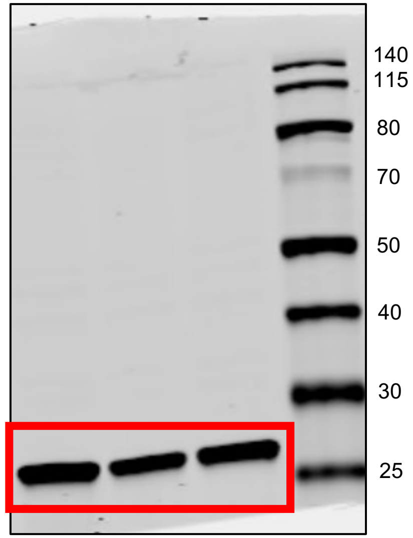

Myc

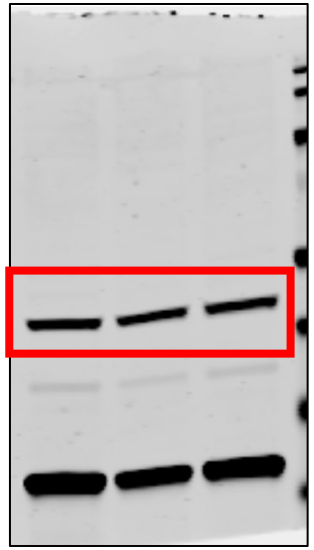

Actin

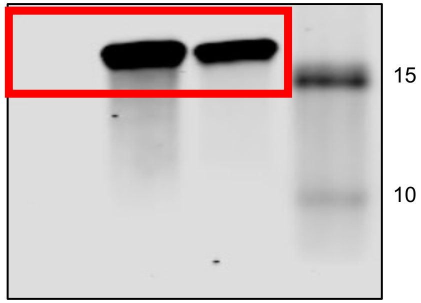

FLAG

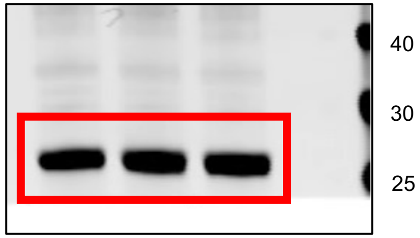

Myc

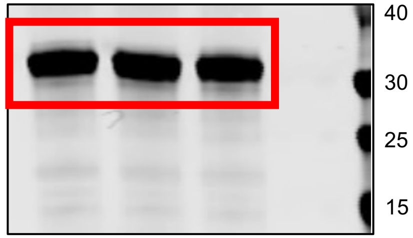

HA

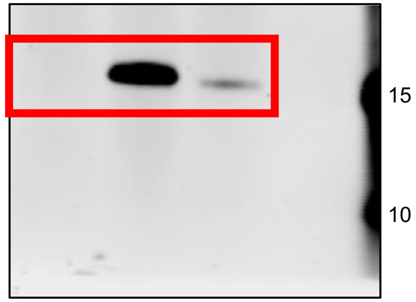

FLAG
